# Hydrophobic Eutectic Solvents for Sustainable Bisphenol A Extraction from Water: Screening and Selection Based on Key Performance Criteria

**DOI:** 10.1021/acsomega.5c07390

**Published:** 2025-09-16

**Authors:** Chiara Carotti, Grazia Isa C. Righetti, Alberto Mannu, Arianna Rossetti, Laura Riva, Gloria Nicastro, Francesco Briatico Vangosa, Carlo Punta, Andrea Mele, Maria Enrica Di Pietro

**Affiliations:** † Department of Chemistry, Materials and Chemical Engineering “Giulio Natta”, 18981 Politecnico di Milano, Piazza Leonardo da Vinci 32, Milano 20133, Italy; ‡ INSTM—Local Unit c/o Politecnico di Milano, Via Mancinelli 7, Milano 20131, Italy

## Abstract

Hydrophobic Eutectic Solvents (HES) are emerging as a promising class of sustainable solvents for water treatment applications, yet their rational selection and performance evaluation remain limited. In this work, we adopted a systematic approach to rank a diverse set of HES, combining terpenes, fatty acids, and trioctylphosphine oxide (TOPO). The screening was based on seven key criteria—viscosity, density, water leaching, pH shift, water uptake, EcoScale score, and chemical stability—aimed at identifying systems with low cross-contamination, high processability, and favorable environmental profiles. Selected HES were then evaluated in liquid–liquid extraction (LLE) using bisphenol A (BPA) as a model compound, with performance assessed via UV–Vis and NMR spectroscopy. The optimized HES, TOPO:menthol, demonstrated an excellent extraction efficiency and very low leaching. Reusability was explored as well as regeneration through back-extraction. Sustainability assessments using the Analytical EcoScale and AGREEprep tools reinforced the viability of the proposed systems. Beyond pollutant removal, this work offers a framework for the molecular-level design and evaluation of HES, advancing their integration into green separation technologies.

## Graphical abstract

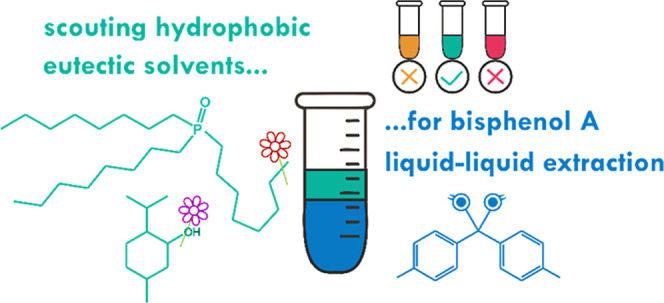

## Introduction

Despite recent worldwide progresses in improving water quality, water bodies remain vulnerable, with emerging contaminants (ECs) constantly added to pollutants’ watchlists.^1^ These synthetic or naturally occurring substances—including pharmaceuticals and personal care products, per- and polyfluoroalkyl substances, and micro- and nanoplastics—pose recognized risks to human health and ecosystems. An additional degree of complexity is the increasing presence of ECs in wastewater, which often lack clear regulations.^2,3^ ECs can be extremely robust in the aquatic environment and often bypass treatment processes designed for solid particles, making them difficult to remove with conventional water and wastewater treatment methods.^3^ These challenges underscore the need for efficient and sustainable technologies for water pollution monitoring and remediation.

A prototypical EC is bisphenol A (BPA), a high production volume (HPV) chemical widely used in manufacturing polycarbonate plastics and epoxy resins.^4^ BPA belongs to the family of endocrine-disrupting compounds (EDCs) and is listed as reproductive toxicants of category 1B and substance of very high concern under the REACH Regulation.^5,6^ It is recognized to be very toxic to aquatic life even in very small quantities (1000–10,000 μg/L) and shows estrogenic effects at concentrations below 1 ng/L.^7^ BPA can infiltrate water environments through multiple pathways, with releases of BPA to the environment estimated to exceed 1 million pounds per year.^4^ While the European Commission has implemented regulations on BPA in surface water (Environmental Quality Standard equal to 0.034 ng/L)^8^ and drinking water (parametric value equal to 2.5 μg/L),^9^ wastewater limits are not uniformly established across all member states.

Current BPA removal strategies include biological, thermal, or electrochemical degradation, adsorption strategies, ozonation, membrane separation processes, and photo- and biochemical-oxidation, each technology characterized by its own merits and demerits.^7,10–12^ Among the water treatment technologies, liquid–liquid extraction (LLE) and its miniaturized version are attractive, thanks to their robustness, cost-effectiveness, and ease of use,^10,13,14^ but suffer from a large consumption of harmful solvents. A possible solution is replacing the toxic volatile organic solvents with Deep Eutectic Solvents (DES) and in particular Hydrophobic DES (HDES).^15,16^ As the nomenclature of DESs is highly debated,^17^ herein, the catch-all term Hydrophobic Eutectic Solvents (HES)^15^ will be adopted.

HES are a subgroup of type III (ionic, typically a quaternary phosphonium/ammonium salt and a hydrogen bond donor HBD)^18^ and type V (nonionic, with only molecular species, the first introduced being composed of menthol and carboxylic acids) DES.^19^ The latter are particularly appealing for their lower viscosity (tens vs hundreds mPa·s) and water solubility (e.g., 0.3–2.5% for menthol:carboxylic acid, 2–7% for systems based on long alkyl chain ammonium salts), both key factors in extraction.^20^ With highly tunable compositions, HES offer wide pollutant compatibility and increased selectivity.^14,21^ Ideally, HES show a perfect combo between ease of preparation (100% atom economy, no purification steps, low energy consumption) and large availability and biodegradability and biocompatibility of most raw materials, leading to low cost and high sustainability.^22^ Albeit eco-safety cannot be guaranteed by definition,^23,24^ prototypical type V HES precursors are bioderived, renewably sourced, and toxicologically well characterized, making them sustainable choices.^25^

All these properties, together with their water-immiscibility, make HES interesting sustainable candidates for water remediation.^26,27^ HES show low detection limits and high extraction efficiencies with low volumes consumed, ensuring simple operation, short extraction time, and no need of additional dispersers. Hydrophobic or quasi-hydrophobic ES have been applied as liquid extractants of BPA from water,^28–34^ food,^35–37^ beverages,^38–42^ blood,^43^ or sludge samples.^44^ There are also reports with HES incorporated in polymers or films for BPA adsorption^45–47^ as well as used as supporting liquid film in hollow-fiber liquid phase microextraction.^48^

Despite promising results, HES development for water treatment is still nascent and often lacks sustainability-focused design. Aiming from one side at expanding the pool of HES suitable for BPA removal and on the other at advancing in their molecular description as extracting agents, we propose here a rational screening of a set of HES obtained by combining terpenes, carboxylic acids, and a phosphine oxide, on the basis on seven criteria, namely, viscosity, density, pH change of the water phase upon mixing, HES leaching to the water phase, water uptake of the solvent upon mixing, EcoScale metrics, and stability. This screening is intended to meet two specific needs. From the one side, we seek extracting agents that are as safe as possible, i.e., with a high EcoScale score and causing low cross contamination, which means in turn characterized by low leaching of the components into the aqueous phase and minimal pH change of water after contact. From the other, it is important to prioritize solvents that can be easier to handle on a larger scale. To this end, HES with low viscosity and large density difference with respect to water are preferred as they increase industrial processability and reduce energy demand. Equally important at a larger scale are a reduced water uptake, as it simplifies the reuse and/or recycling of the HES, and the absence of significant change and/or degradation of the solvent over time, as it enables its storage. The top-performing HES, composed of trioctylphosphine oxide (TOPO) and menthol (Men), is then used to optimize the LLE protocol of the model compound BPA from water. TOPO is a lone hydrogen bond acceptor known for its ability to form HES with strong deviation from thermodynamic ideality.^17^ Its excellent complexation ability has been largely exploited, by dissolving it in organic solvents for the extraction of inorganic and organic species including phenolic compounds,^49^ and TOPO-containing HES have been already reported as extracting agents of metal ions and organic acids.^49–54^ However, to the best of our knowledge, only a very recent paper reports the use of TOPO as a surfactant to modulate the extraction ability of a ternary menthol:nerol:formic acid ES in the microextraction of bisphenols in water.^48^ After LLE optimization at the lab scale with TOPO:Men, selected HES are compared in terms of both extraction efficiency and leaching into water. Established green metrics are applied for a sustainability assessment of the whole process.^55,56^ Finally, the reuse of the same BPA-rich HES phase in consecutive extractions is carried out for the best-performing HES, followed by solvent regeneration via back-extraction and its reuse.^31^

## Experimental Section

### Preparation of Hydrophobic Eutectic Solvents

Four terpenes (camphor, Cam; carvone, Car; menthol, Men; thymol, Thy), five carboxylic acids (hexanoic acid, HexA; octanoic acid, OctA; decanoic acid, DecA; dodecanoic acid, DodA; oleic acid, OleA), and a phosphine oxide (trioctylphosphine oxide, TOPO) (Figure 1 and Table S1) were combined to prepare a set of 13 HES, listed in Table 1. HES were prepared by the heating method: the two components were weighted into a screw-capped sample vial using an analytical balance (OHAUS Explorer, precision = 0.1 mg), mixed at the desired mole fractions, and then heated under stirring at 50 °C, using a thermoregulated bath (VFT Fuzzy Logic Thermoregulator, Fisherbrand), until a homogeneous liquid was formed and then stirred in the liquid state for almost 30 min. The eutectic composition—or a close one—as described in the literature from the solid–liquid equilibrium diagrams was used for all HES,^17,53,57–64^ but Men:HexA. In the absence of a solid–liquid equilibrium diagram, the latter was prepared at a 1:1 molar ratio, following previous extraction studies with this system.^65,66^ The selection includes both HDES with negative deviation from thermodynamic ideality (i.e., Thy:Cam, TOPO:Men, and TOPO:DodA) and ideal or close-to-ideal HES (Men:Cam, Men-based HES with long-chain carboxylic acids, Car-based HES, and all-fatty-acid-based ones).

**Figure 1:**
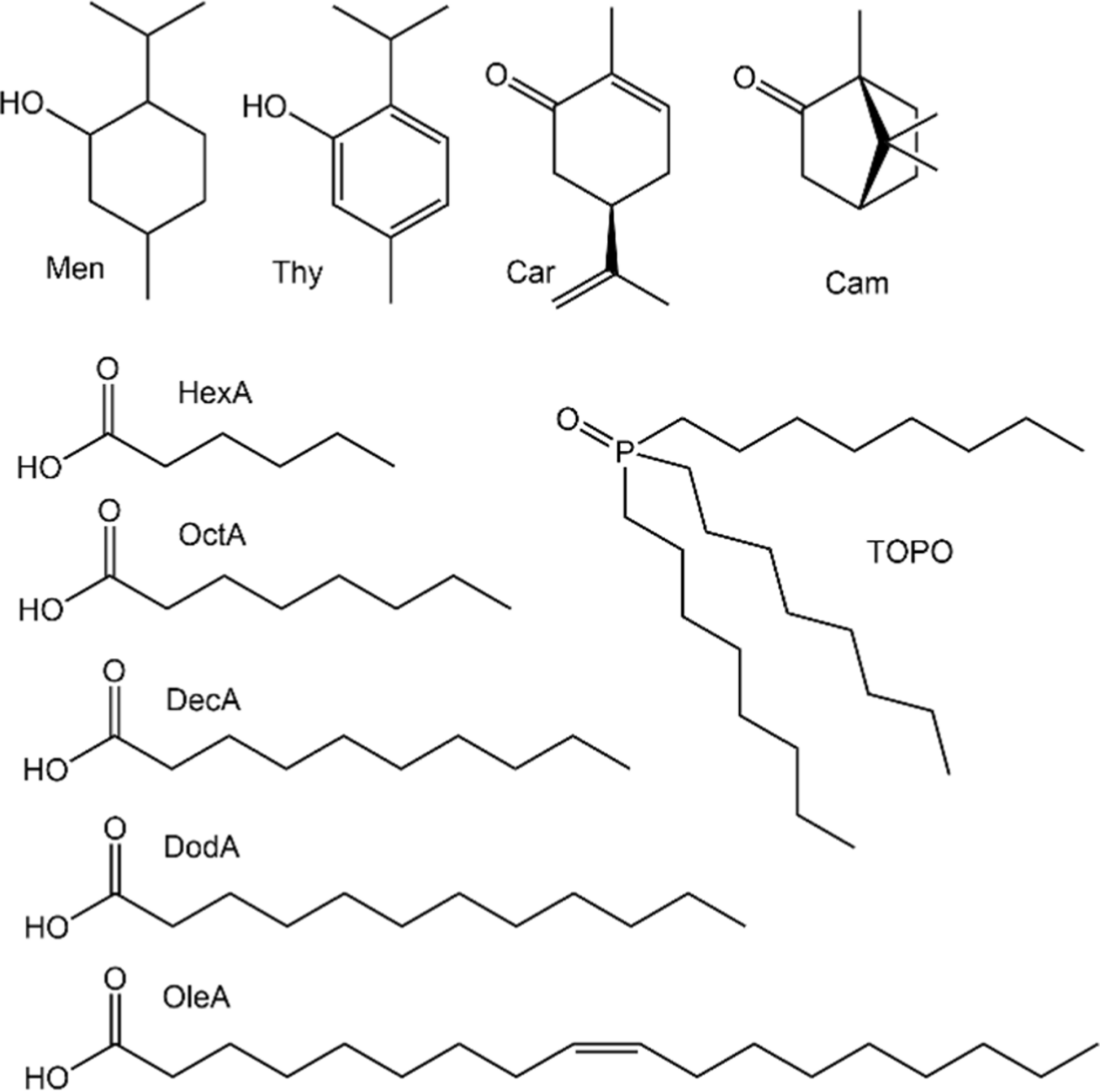
Molecular structures of the HES components: menthol (Men), thymol (Thy), camphor (Cam), carvone (Car), hexanoic acid (HexA), octanoic acid (OctA), decanoic acid (DecA), dodecanoic acid (DodA), oleic acid (OleA), and trioctylphosphine oxide (TOPO).

**Table 1:** HES Used in This Work

component 1	component 2	mole ratio	abbreviation
menthol	camphor	2:1^a^	Men:Cam
thymol	camphor	1:1^b^	Thy:Cam
menthol	hexanoic acid	1:1^c^	Men:HexA
menthol	octanoic acid	1:1^d^	Men:OctA
menthol	decanoic acid	2:1^d^	Men:DecA
menthol	oleic acid	2:1^e^	Men:OleA
trioctylphosphine oxide	menthol	1:2^f^	TOPO:Men
trioctylphosphine oxide	dodecanoic acid	1:1^g^	TOPO:DodA
carvone	menthol	3:1^h^	Car:Men
carvone	octanoic acid	4:1^h^	Car:OctA
carvone	decanoic acid	9:1^h^	Car:DecA
octanoic acid	decanoic acid	3:1^i^	OctA:DecA
octanoic acid	dodecanoic acid	3:1^j^	OctA:DodA

a From refs 57,58 .

b From ref 57.

c From refs 65,66 .

d From refs 59,60 .

e From ref 61.

f From ref 17.

g From ref 53.

h From ref 62.

i From ref 63.

j From ref 64.

### Density and Viscosity of Neat HES

Density was measured in triplicates at room temperature by gravimetry: known volumes of each HES were added into a graduated cylinder, and the weight was recorded. Viscosity was measured in triplicate at 25 °C, using an Anton Paar MCR502 rheometer with a cone–plate configuration (50 mm diameter, 1° angle, and 99 μm truncation). Temperature was controlled with a Peltier plate and hood system (H-PTD P-PTD 200). Shear rate was varied following a logarithmic ramp profile, ranging from 10 s^–1^ to 100 s^–1^, with data points collected at a density of 15 points per decade. Viscosity values were determined through the linear regression of the shear stress dependence on the shear strain rate. Viscosity measurements were also performed in duplicate after one month under the same operating conditions. Results are reported in Table S2.

### HES Leaching, pH Change, and Water Uptake after Mixing with Water

2.0 mL of deionized water and 1 g of HES were weighed in an 8 mL vial and mixed under stirring on a hot plate magnetic stirrer (Velp Scientifica Arex 5) at 25 °C and 900 rpm for 1 h. After mixing, HES and water phase were centrifuged (Orma Scientific CN45) at 4000 rpm for 30 min and separated. Leaching of HES into the water phase was measured via quantitative NMR spectroscopy (vide infra), and the results are reported in Table S3. The solubilities of the pure precursors are also given in Table S4 for comparison. The pH of the water phase after mixing with the HES was measured using a Mettler Toledo compact pH/ion meter (S220), previously calibrated using standard solution at pH 4.0 and 7.2. The water content was measured for each HES before and after mixing with water using a Karl Fisher Coulometric Moisture Titrator (KEM MKC-720) previously calibrated with the reference material (HYDRANAL—CRM Water Standard 1.0, water content 1 mg/g = 0.1%, purchased from Honeywell). Results are reported in Table S5.

### EcoScale Metrics

EcoScale for each HES preparation was calculated as follows:

1 EcoScale=100−∑penaltypoints

The penalty points (PPs) were assigned according to the table reported in the original publication of Van Aken et al.,^67^ which considers six general parameters: (1) yield; (2) price of the reagents; (3) safety; (4) technical setup; (5) temperature/time; and (6) workup and purification. The estimation of reagent costs (parameter 2) was performed by considering the current price found in the online catalogue (last accessed in June 2025) of the generic supplier Sigma-Aldrich for a given pack size (100 g). Penalty points on safety (parameter 3) were assigned based on the hazard profile of the compounds, as gathered by inspecting the safety data sheets (SDS) of the actual supplier: N (dangerous for the environment), T (toxic), F (highly flammable), E (explosive), T+ (extremely toxic), and F+ (extremely flammable). For each binary mixture, the total PPs are calculated as the sum of the PPs of the two precursors. Results are reported in Table S6.

### Liquid–Liquid Extraction of Bisphenol A

An aqueous solution of BPA at a concentration of 100 ppm was prepared gravimetrically. The BPA-contaminated water and the HES were put into contact in a HES:water ratio of 1:2 (w/w, with a total sample size of 3 g) and stirred at 900 rpm in vials of 8 mL, at 25 °C and atmospheric pressure. Centrifugation was then used to achieve proper phase separation (30 min, 4000 rpm). Samples of the aqueous phase were separated, and the quantification of BPA as well as the HES precursor leaching was carried out by UV–vis spectrophotometry and ^1^H NMR spectroscopy (vide infra). HES samples were also used for direct BPA quantification via NMR spectroscopy. Each LLE was performed in triplicate.

The extraction efficiency (EE) for BPA removal was calculated from the residual concentration of contaminant in the water phase (as measured by either UV–vis or NMR) according to eq 2

2 $$EE\left[\%\right]=\frac{C_{BPA}^{i}-C_{BPA}^{f}}{C_{BPA}^{i}}\cdot 100$$

where *C*_BPA_^i^ and *C*_BPA_^f^ are the initial and final concentrations of BPA in the water phase, respectively.

The leaching of the HES component(s) in the water phase (Leach­(*i*), *i* being the HES component) was calculated from the residual concentration of the given species in the water phase (as measured by NMR) according to eq 3

3 $$Leach\left(i\right)\;\left[\%\right]=\frac{{m\left(i\right)}_{water}^{f}}{{m\left(i\right)}_{HES}^{i}}\times 100$$

where *m*(*i*)_water_^f^ is the final mass (in mg) of each HES component *i* in the water phase after extraction and *m*(*i*)_HES_^*i*^ is the mass (in mg) of the same component *i* in the HES before the extraction.

TOPO:Men was used for LLE optimization, fixing both the HES:water ratio (1:2 w/w, with a total sample size of 3 g) and the temperature (RT) and varying the contact time (10 min vs 1 h) and the mixing procedure (stirring vs no stirring, with or without centrifugation). Three more HES from the preliminary screening were evaluated using the previously optimized protocol (10 min, RT, stirring, and centrifugation), namely, TOPO:DodA, Men:OleA, and OctA:DodA.

### UV–Vis Measurements

UV–vis absorption spectra were acquired using an Onda Scan spectrophotometer UV31, within a wavelength range of 195–1100 nm, with a step size of 1.0 nm. The samples were placed directly into quartz cuvettes with a 1 cm path length. Characteristic BPA absorbance was detected at a wavelength of 276 nm. A standard calibration curve was built in deionized water in the range 1.5–100 ppm (*R* ^2^ = 0.9988, Figure S1).

Due to the HES leaching, a matrix-matched calibration curve was also implemented. Standard BPA solutions in the range 0.78–50 ppm were prepared by diluting water contaminated with BPA at known concentrations with water after a blank extraction test with neat HES (hence potentially containing leached HES components) (*R* ^2^ = 0.9971, Figure S1).

### NMR Measurements

Spectra of neat HES were acquired on a Bruker NEO 500 MHz spectrometer, operating at a magnetic field strength of 11.7 T, equipped with a BBFO probe-head. Neat HES were transferred to standard 5 mm NMR tubes, equipped with a coaxial insert containing DMSO-*d*_6_ and tetramethylsilane (TMS), for lock and chemical shift reference, respectively. ^1^H NMR spectra were acquired on freshly prepared HES, HES after contact with water, and pure HES one month after preparation. For HES composed of Men and carboxylic acids, additional ^1^H NMR spectra were acquired 3, 5, 8, and 10 months after preparation. All spectra were recorded at 25 °C using 64k data points, 8 scans, and a 1 s relaxation delay. Spectra were processed by using an exponential line broadening (LB) of 0.3 Hz and manual phase correction.

Quantitative NMR (qNMR) spectra of water samples after blank tests (mixing with water) and LLE experiments were acquired on a Bruker Avance III 400 MHz spectrometer, operating at a magnetic field strength of 9.4 T, equipped with a PABBO probe-head. Samples were prepared by adding 500 μL of the aqueous phase after the LLE experiment into a standard 5 mm NMR tube, already filled with 50 μL of a TSP (trimethylsilylpropanoic acid) aqueous solution at a known concentration (11.6 mM), which served as an internal reference. Spectra were recorded at 300 K with 64k data points, 128 scans, and a 4 s relaxation delay. Water presaturation was achieved using the standard NOESY presaturation scheme (Bruker pulse program noesypr1d). Spectra were processed using an exponential line broadening (LB) of 2 Hz, manual phase correction, and automatic baseline. Selected signals corresponding to different protons of each species (BPA, HES precursors) were integrated, and concentrations were calculated as follows:

4 $$C_{sample}=\frac{I_{sample}\times N_{ref}}{N_{sample}\times I_{ref}}C_{ref}$$

where *C*_sample_ and *C*_ref_ are the unknown concentration of the analyte (BPA or HES species) and the known concentration of TSP (1.05 mM) in the NMR sample, *I*_sample_ and *I*_ref_ are the integrated areas of the proton signals for the analyte and TSP, and *N*_sample_ and *N*_ref_ are the number of the protons associated with each signal for the analyte and TSP. The LOD and LOQ were calculated at signal-to noise (S/N) ratios of 3 and 9, respectively (Figure S2).

Quantitative NMR (qNMR) spectra of the HES phase after LLE were acquired on a Bruker NEO 500 MHz spectrometer, operating at a magnetic field strength of 11.7 T, equipped with a BBFO probe-head. Samples were prepared by adding 500 mg of the HES phase after the LLE experiment into a standard 5 mm NMR tube, already filled with 50 mg of a TSP (trimethylsilylpropanoic acid) solution in HES at a known concentration (11.6 mM), which served as an internal reference. Spectra were recorded at 50 °C with 64k data points, 128 scans, and a 5 s relaxation delay. Spectra were processed using an exponential line broadening (LB) of 2 Hz, manual phase correction, and manual baseline. Selected signals of BPA were integrated, and concentrations were calculated using eq 4.

### Reuse and Regeneration of HES

Scale-up tests were performed with TOPO:Men to evaluate the possible reuse of the solvent and its extraction performance during several extraction cycles. In the first extraction cycle, TOPO:Men (10 g) was brought into contact with the 100 ppm of BPA solution (20 mL), according to the optimized protocol. The HES, after extraction and separation, was reused in a second extraction cycle with a fresh BPA solution under the same conditions. The procedure was repeated for 10 cycles, scaling the total volume at each cycle in order to keep a constant 1:2 HES:water ratio. The HES phase separated at the 10th cycle was regenerated via a back-extraction of BPA using NaOH 1 M solution, adapting a published protocol:^31^ 2 mL of the final HES phase was put in contact with 2 mL of NaOH 1 M solution at 303 K, 800 rpm, for 6 h. Both the HES phase and the basic solution were analyzed via NMR spectroscopy. The regenerated HES phase was used in a new LLE experiment under the usual conditions.

## Results and Discussion

### HES Screening

For application in water treatment, the ideal HES system should be stable and as sustainable as possible and should ensure a sharp separation interface after contact with water, with cross contamination as low as possible. Seven criteria were then considered for the HES screening: viscosity, density, pH change of water phase upon mixing, HES leaching to the water phase, water uptake of the HES upon mixing, EcoScale metrics, and chemical stability. Low viscosity (η) and large density difference (Δρ) with respect to water are essential to simplifying phase separation. Viscosity values below 100 mPa s and density differences of at least 50 kg m^–3^ were previously considered as suitable thresholds for separation purposes.^68^ In our case, viscosity ranged from 3 mPa s for Car-based HES to 40–50 mPa s for TOPO-based ones (Figure 2a and Table S2), making all HES a viable option. All systems are less dense than water, with Thy:Cam 1:1 and Car-based HES showing density values unacceptably close to 1 g/mL (Δρ ≈ 20 kg m^–3^ for Thy:Men and Δρ in the range 30–50 kg m^–3^ for Car-based HES) and hence discarded (Figure 2a and Table S2). The nine remaining HES showed satisfactory Δρ values between 100 and 65 kg m^–3^.

**Figure 2:**
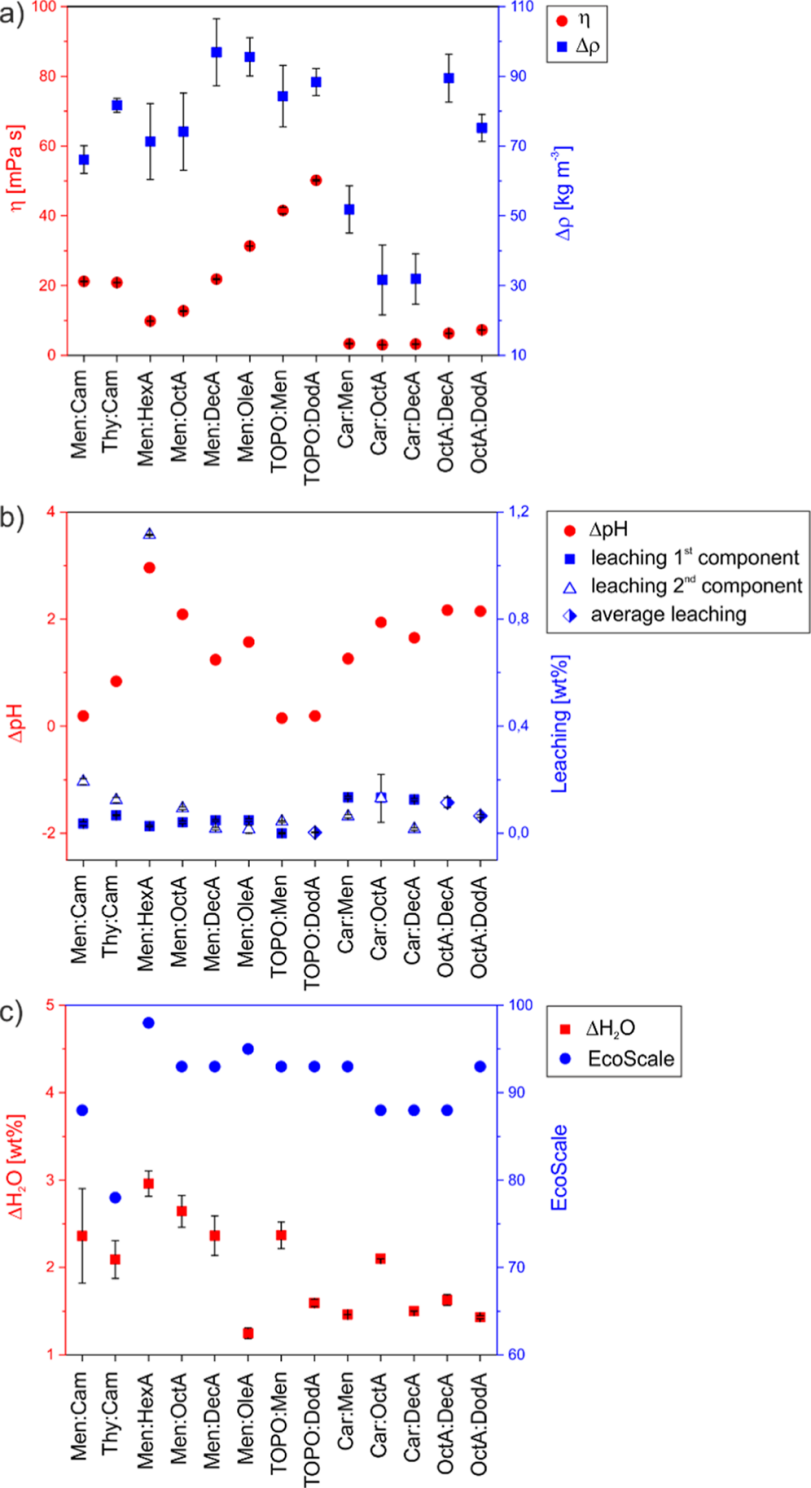
Criteria for HES screening: (a) viscosity (η, to minimize) and density difference with respect to water (Δρ, to maximize); (b) pH change of the water phase after mixing with HES (ΔpH, to minimize) and HES leaching in the water phase after mixing (to minimize); and (c) water uptake in HES after mixing with water (ΔH_2_O, to minimize) and EcoScale score for HES preparation (to maximize).

For application in water remediation, reducing the cross contamination due to leaching of HES precursors is of utmost importance.^26^ To this end, the concentration of leached compounds in water (Leach­(*i*)) was measured via NMR spectroscopy by integrating the corresponding signals in the ^1^H NMR spectra of the water phase after a blank test consisting of 1h of stirring at 25 °C with a HES-to-water ratio of 1:1. Given the different natures of the precursors, this parameter varies quite markedly over the composition (Figure 2b and Table S3). Moreover, the partitioning of the two HES components over the HES-rich and water-rich phases appears to be rather asymmetric and random and is mainly driven by the individual water solubility (Table S4). Due to its short alkyl chain, HexA leaching was unacceptably high (above 1 wt %). The precursors Cam and Car as well as the HES OctA:DecA also exhibited significant migration to the water phase, with a final concentration above 0.1%. The remaining six HES (Men:OctA, Men:DecA, TOPO:Men, TOPO:DodA, Men:OleA, OctA:DodA) gave leaching values lower than 0.1 wt %, which was considered an acceptable threshold and is indeed much better than that of other type III hydrophobic HES.^18^ Although strongly influenced by experimental conditions (contact time, relative volumes, and temperature, among others), these values provide a consistent ranking of the selected set of HES for the desired application and align well with values previously reported in the literature.^64,68^ In all cases, the leaching was low enough not to alter the eutectic compositions, as confirmed by the integral ratio of the NMR signals in the ^1^H spectra run after mixing with water (Figures S3–S15).

A further indirect experimental evidence of HES leaching was obtained by measuring the pH change (ΔpH) of the water phase after the blank test (Figure 2b and Table S5). This parameter is severely affected by the inherent nature of the component, with carboxylic acids significantly decreasing the resulting pH (ΔpH in the range 1.2–3.0 for all HES containing a carboxylic acid, the only exception being TOPO:DodA, with ΔpH = 0.19). For the remaining HES, Men:Cam and TOPO:Men exhibited acceptable ΔpH of 0.19 and 0.15, respectively. In a complementary way, the water uptake (ΔH_2_O) in the HES phase after the blank test was also measured via Karl Fischer titration and is reported as the difference between the water content (in wt %) after contact with aqueous phase and the initial water content (Figure 2c and Table S5). As already observed for other type V HES, all HES show low water content upon mixing with water (1.3–3.1 wt %),^64,68^ with Men:HexA showing the highest water uptake (3 wt %).

HES chemical stability was evaluated by measuring the macroscopic viscosity as well as by recording the ^1^H NMR spectra of the same samples after one month of storage in closed vials/tubes at RT. Viscosity values remained largely unchanged (Table S2), and most HES showed no degradation and/or noticeable changes in the ^1^H NMR spectra (Figures S3–S15). The four systems composed of Men and a carboxylic acid showed an extra signal corresponding to the ester formation (Figures S16–S19), as already observed for mixtures of Men and OctA,^69^ and reasonably occurring in a mixture of organic acids and alcohols.^70^ Even though an extended evaluation of the impact of HES esterification on the extraction performance is beyond the scope of this study, the esterification reaction was considered here as an unwanted degradation and was monitored over time by integrating the signal at 4.3 ppm (assignable to M1 of the menthol ester) and the signal at 3.0 ppm (M1 of free Men). While the ester signal is undetectable in the freshly prepared sample, it appears already after one month and progressively raises after 10 months (Figure S20). Shorter alkyl chains such as in HexA and OctA lead to higher esterification rates, with the menthol ester reaching up to 14% after 10 months. In the case of DecA and OleA the esterification was reduced, with 6% and 4% of menthol ester in the mixture after 10 months, respectively.

To introduce a first sustainability descriptor in the screening process, EcoScale analysis was applied on the prepared HES (Table S6). In terms of EcoScale metrics,^67^ the heating introduces 2 penalty points (PPs). The parameters Yield (100%), Technical Setup (common), Workup, and Purification (none) do not introduce penalties. All HES are inexpensive except Men:OleA, for which 3 PPs were added (please refer to the Experimental section for details on cost estimation). According to the hazard profile reported in the corresponding SDS, no penalties are considered for Men, DodA, HexA, and OleA while 5 PPs are introduced for TOPO, OctA, and DecA due to their moderate aquatic hazard (H411 – H412). Ten PPs are introduced for Cam as it is classified as a flammable solid (H228) and associated with acute toxicity (H332). Ten PPs are also introduced for Thy due to its moderate aquatic hazard (H411) and acute toxicity (H302). The sum of individual penalties is then between 2 (Men:HexA, EcoScale = 98) and 22 (Thy:Cam, EcoScale = 78) (Figure 2c and Table S6). All HES are ranked as excellent in the EcoScale metrics (score >75), even though further investigation into their toxicity and ecotoxicity should be performed and caution should to be paid in the HES selection.

The combination of the seven criteria led us to discard the HES with the largest leaching (Men:Cam, Men:HexA, and all Car-based HES), prioritizing cross-contamination as the key criterion. Thy:Cam was also discarded due to its density value, being too close to water, and the lowest EcoScale score. TOPO:Men and TOPO:DodA stand out as the most promising HES, showing Δρ of 85–88 kg m^–3^, good stability, high EcoScale score, and especially a very low leaching, confirmed by the small ΔpH. Among them, the less viscous TOPO:Men was selected for LLE optimization. Among the remaining Men-containing HES, Men:OctA showed the highest leaching, while Men:DecA and Men:OleA exhibited a lower leaching and an overall comparable performance. Men:OleA was then selected as a candidate for BPA removal due to the higher EcoScale value and minimum ester formation. Finally, the two all-fatty acids HES showed both very low viscosity and acceptable density values. OctA:DodA was then selected due to the reduced cross contamination.

### Liquid–Liquid Extraction of Bisphenol A

TOPO:Men emerged as the most promising HES in the screening process due to its large density difference from water, low viscosity, high stability, favorable EcoScale score, and minimal leaching. It was therefore chosen as a model HES to be tested in the LLE of BPA from water. Moreover, TOPO shows excellent complexation ability, and TOPO-based HES have been already reported as effective extractants of metal ions and organic acids,^49–54^ supporting its potential for BPA extraction. Three operating conditions were sequentially evaluated, trying when possible to reduce energy consumption: contact time (1 h vs 10 min), stirring (900 rpm vs none), and centrifugation (4000 rpm for 30 min vs none).

Before going through a detailed description, it is worth mentioning that analytical quantification may be challenging. The extraction efficiency for the removal of a contaminant is routinely calculated by measuring the residual contaminant concentration in the water phase. Two opposite approaches were selected here: UV–vis and NMR spectroscopy. The former is characterized by wide accessibility at a low cost and enables a rapid and straightforward quantification of the analyte without sample manipulation or extensive personnel training. These advantages come at the cost of high sensitivity to matrix effects, which can severely bias the outcomes. This is evident looking at the UV–vis spectrum of a water solution after mixing with HES, i.e. the black test described in the experimental section: a small yet detectable absorption peak appears at the BPA absorbance wavelength of 276 nm (leachate in Figure S1a). This introduces a matrix effect in BPA quantification, as shown in the comparison of the absorption spectra of an aqueous solution of BPA at 25 ppm with an aqueous solution at 25 ppm of BPA contaminated with HES TOPO:Men (Figure S1a). A matrix-matched calibration curve was then implemented (Figure S1b), attempting to overcome this issue by considering the contribution of HES leaching to the intensity of the absorption peak of BPA at 276 nm. Note, however, that HES leaching a priori depends on the mixing conditions (e.g., time, temperature, HES/water ratio), making it hard to set a general protocol.

NMR spectroscopy is, on the other hand, a powerful tool that allows for the individual quantification of single compounds in a complex mixture without physical separation. Hence, it is then inherently much less susceptible to matrix effects as long as at least one isolated peak belonging to each compound can be identified. Additionally, the opportunity to quantify each species by integrating its specific NMR signal in the ^1^H spectrum enables the quantification of both the residual contaminant (BPA here) and HES species after leaching into the water phase. This can be easily seen in Figure 3b, which displays a representative ^1^H NMR spectrum of the water phase after LLE with TOPO:Men. Note that more than one signal corresponding to Men protons are isolated enough to allow proper integration, providing a tool to assess intravariability. However, NMR quantification requires minimal sample preparation, is more energy-consuming compared with UV–vis, and is typically characterized by lower sensitivity (higher limits of detection) and longer experimental time. The LOQ and LOD with our hardware were found at 3.125 ppm (S/N ratio equal to 9) and 1.56 ppm (S/N ratio equal to 3), respectively (Figure S2). Advantages and drawbacks of the two quantification methods are compared in Table S7.

**Figure 3:**
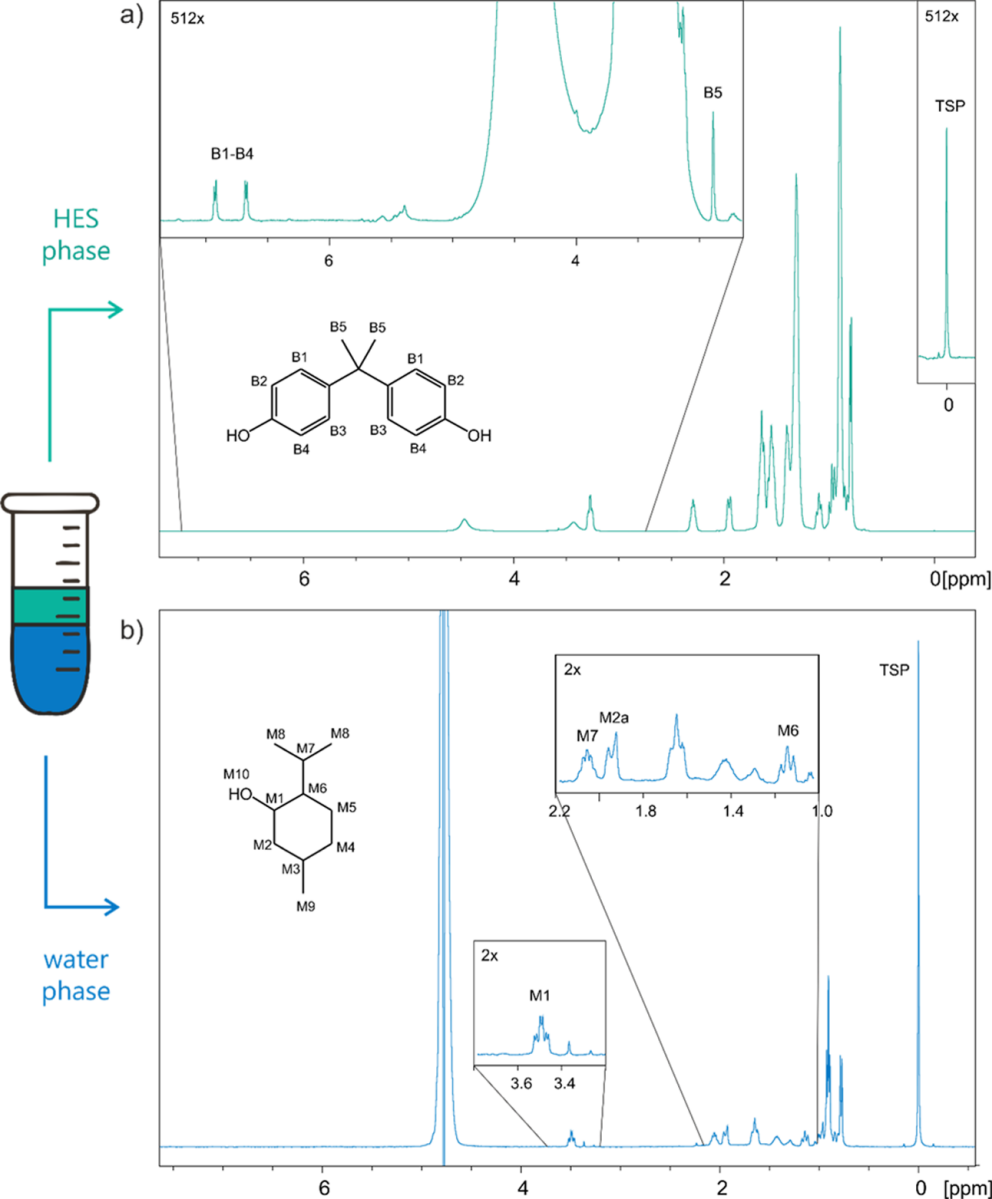
Representative ^1^H NMR spectra of the (a) HES phase and the (b) water phase, after extraction of BPA with the HES TOPO:Men (10 min stirring at rt, followed by 30 min centrifugation). Numbering is given for selected signals used for quantification.

The indirect calculation of the extracted BPA by the difference between the initial concentration and residual concentration after LLE is more susceptible to error than a direct quantification in the HES phase, given the highest concentration expected in the latter. Therefore, we propose an additional NMR quantification protocol for the HES phase (see the Experimental section for details) to be used to validate and complement data obtained for the water phase. Figure 3a shows a representative ^1^H NMR spectrum of TOPO:Men after LLE, where the BPA signals are well distinguishable. It should be noted that measurements were performed at 50 °C to improve the inherently low resolution of ^1^H NMR spectra obtained for relatively viscous neat liquids, and the spectra required manual processing to ensure reliable integration and quantification. Overall, the method offers a useful complementary tool for direct quantification; however, some of the limitations of qNMR become more pronounced, particularly the need for careful sample preparation and advanced operator training, and the errors are quite large.

UV–vis spectra of the water phase (using the matrix-matched calibration curve) and NMR spectra of both water and HES phase were recorded here and analyzed to calculate EE. All results are reported in Tables S8–S11, with HES leaching obtained from NMR spectra of the water phase (Tables S8 and S10). Figure 4a and Tables S8 and S9 summarize the results obtained for the one-step extraction of BPA using HES TOPO:Men under different operating conditions. It can be observed that the efficiency significantly decreased when no stirring is applied (complete extraction for “1 h stirring” vs almost 60% for “1 h no stirring”), with no remarkable effect on HES leaching (roughly 0.1% in both cases). This outcome suggests that a proper mass transfer due to vigorous stirring is essential for the extraction process,^29^ but does not enhance cross-contamination. Looking at the contact time, the extraction of BPA occurs rapidly, and the equilibrium is reached already after 10 min, in line with previous observations.^29,32^ Such fast kinetics is not only highly desirable from a sustainability viewpoint but also beneficial in terms of HES leaching, which is slightly reduced (ca. 0.10% for “1 h stirring” vs 0.08% for “10 min stirring”). Aiming at reducing the number of steps in the procedure and the energy-consuming operations, centrifugation was omitted, allowing the two phases to separate by gravity overnight. Even though the EE remains satisfactory (complete extraction in both “10 min stirring” and “10 min stirring no centrifugation”), the HES leaching slightly increased, plausibly due to the long contact time between the two phases.

**Figure 4:**
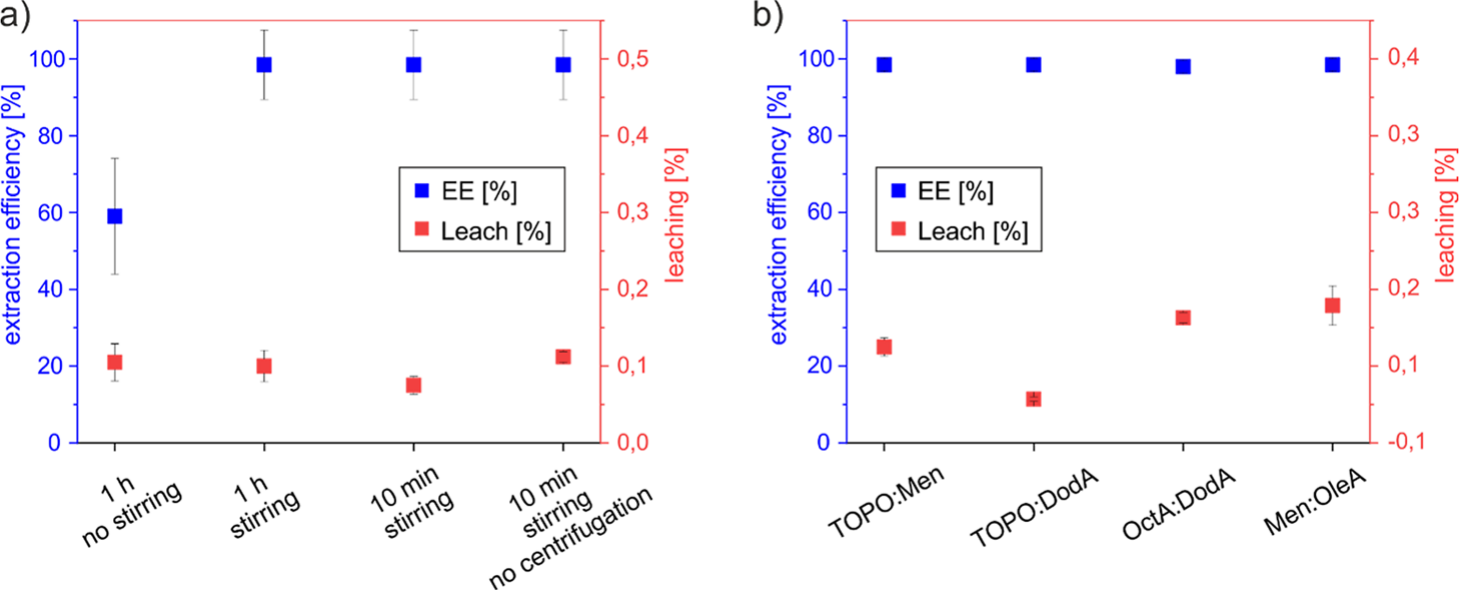
(a) BPA extraction efficiency (EE) and HES leaching using TOPO:Men under various experimental conditions, and (b) comparison of four HES under the best extraction conditions.

Optimal conditions for the following LLE experiments were then set to 10 min, stirring, and centrifugation. The four selected systems from the preliminary screening (TOPO:Men, TOPO:DodA, OctA:DOdA, and Men:OleA) were compared under the optimized protocol, evaluating both the BPA extraction efficiency and the leaching percentage into water (Figure 4b and Tables S10 and S11). All HES demonstrate excellent EE, with complete extraction for three of them (TOPO:Men, TOPO:DodA, and Men:OleA) and a barely detectable BPA residual signal in the water phase for OctA:DodA. Notably, the leaching data mark a difference between the four HES, with OctA:DodA and Men:OleA showing the highest water leaching (0.113 and 0.129 wt %, respectively), TOPO:Men showing an intermediate value (0.075 wt %), and TOPO:DodA showing a desirable very low leaching (0.007 wt %). This trend is consistent with the screening results and reflects the water solubility of the pure components.

Comparison of the UV–vis and NMR results (Tables S9 and S11) reveals numerical discrepancies, attributable, at least in part, to the high sensitivity of UV–vis to matrix effects arising from HES leaching. While matrix-matched calibration curves can be implemented (Figure S1), a case-by-case calibration would be required to increase accuracy, thus undermining the key advantages of UV–vis, namely, speed and ease of use. Nevertheless, both techniques yield consistent trends: the poorest EE is observed without stirring, whereas good performance is maintained under other conditions. The intentional use of two contrasting techniques underscores their respective advantages and limitations in the context of BPA quantification, demonstrating that both, within their inherent limitations, can ultimately identify optimal HES and extraction protocols.

### Sustainability Assessment

In response to the growing emphasis on Green Analytical Chemistry (GAC),^71^ we applied available assessment tools to evaluate BPA removal via HES as proposed here.

An initial analysis was conducted using the Analytical EcoScale approach introduced by Gałuszka et al., which extends the previously established EcoScale concept,^55^ considering parameters related both to reagents and processes. Although this metric was originally designed for assessing the sustainability of analytical methods in quantification processes, it is adapted here to the LLE protocol and the different HES. To this end, we accounted for all steps, starting from HES preparation to contaminant quantification.

Reagent penalty points (PPs) were calculated based on the Global Harmonized System (GHS) pictograms and associated signal words specified in the safety data sheets (SDS) of the actual supplier (Table S12). Each hazard pictogram corresponds to one PP. If a chemical is labeled with the signal word “Danger” (as for OctA, DodA, and TOPO), the number of hazard pictograms is multiplied by two, whereas for chemicals labeled “″Warning” (i.e., Men), the pictogram count remains unchanged. When no precautionary statement is required (no hazard pictogram nor signal word), no PP is added (i.e., OleA). The total PPs for each binary mixture are determined by summing the PPs of both precursors (Table S13). Since the amount of reagents used in all cases is below 10 mL or 10 g, the final hazard PPs remain unadjusted (multiplied by 1).

Instrument PPs were determined by accounting for the actual energy consumption of each laboratory device (calculated considering power in watts, usage hours, and number of samples) during each procedural step. This includes using the hot plate for HES preparation (30 min) and extraction (10 min), the centrifuge for separation (30 min), and either UV–vis or NMR for quantification (Table S13). No additional PP was assigned for “Occupational Hazard” since no hazardous vapors or gases were emitted. At this stage, no recycling is considered, then the amount of waste is in the range 1–10 mL (3 PPs) and no treatment is performed (with a cost of 3 PPs).

Considering the “Instrument” section, for LLE with 10 min stirring and centrifuge, 7 PPs are accounted when UV–vis is used as the analytical technique, while 2 more PPs need to be added when NMR measurements are used (9 PPs). The difference between the 4 HES is only related to the reagent hazard, which range from 1 PP (Men:OleA) to 4 PPs (TOPO:DodA and OctA:DodA). Overall, all systems are ranked as excellent in the Analytical EcoScale metrics (score >75), with scores decreasing in the order Men:OleA > TOPO:Men > TOPO:DodA = OctA:DodA (Table 2).

**Table 2:** Analytical EcoScale Evaluation in the Scenario of the Optimized LLE Protocol (10 min Stirring and Centrifugation) and UV–Vis Quantification, Using Four Selected HES without HES Recycling, as Well as Using TOPO:Men with HES Recycling

	reagent PPs	energy PPs	occupational hazard PPs	waste PPs	analytical EcoScale
TOPO:Men	3	1	0	6	90
TOPO:DodA	4	1	0	6	89
Men:OleA	1	1	0	6	92
OctA:DodA	4	1	0	6	89
TOPO:Menwith recycling	3	1	0	1	95

In an attempt to provide a refined output that includes the sample preparation step as addressed in the principles of Green Sample Preparation (GSP), we also applied the Analytical GREEnness metric for sample preparation (AGREEprep).^56,72^ This advanced metric tool converts the ten GSP principles in scores and provides an easy-to-read pictogram mapping the degree of compliance of all evaluated criteria. Already applied to assess the environmental impact of DES-based extraction processes with a special attention on sample preparation,^43,73,74^ its application to our small-scale LLE approach has to be seen not as a final evaluation but a way to detect the strength and weaknesses of the method. We consider here the total volume as the whole sample (3 mL) and calculated the scores for the following 4 steps: HES preparation (1 g, 30 min heating, 50 °C), extraction (1 g HES + 2 mL water, 10 min stirring, rt), centrifugation (30 min, 4000 rpm), separation (manual, pipet). Figure 5a–d shows the AGREEprep pictograms obtained for the LLE methodology proposed in this work when UV–vis is used for quantification (see Supporting Information for a more extended description). The segments with lower scores reflect the lab scale of our LLE strategy, with significant amount of waste (criterion 4, where also single-use glassware were considered), a limited number of samples treated in 1 h (criterion 6) and all manual operations (criterion 7). Even though NMR quantification is more energy consuming than the UV–vis counterpart, decreasing the final score of a fraction 0.5–0.6 (Figure S21), we stress here that the nonselectivity of UV–vis quantification may compromise results in specific cases and the choice of the quantification technique needs careful consideration. While Men and the long-chain carboxylic acids OctA, DodA and OleA are considered as sustainable, TOPO is not, thus decreasing the score for TOPO:Men and TOPO:DodA (criterion 3). Similarly, differences between the four systems come also into criterion 2 (where precursors considered as harmful to aquatic life are included) and criterion 10 (where GHS pictograms are considered), with Men:OleA and TOPO:Men at the extremes (0.61 and 0.44 in case of UV–vis analytical quantification, respectively).

**Figure 5:**
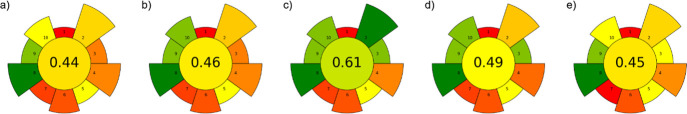
Result of AGREEprep assessment of the LLE procedure for BPA extraction using (a) TOPO:Men 1:2, (b) TOPO:DodA 1:1, (c) Men:OleA 2:1, (d) OctA:DodA 3:1, and (e) TOPO:Men 1:2 with HES recycling, when UV–vis is used as the analytical method.

Men:OleA exhibits the highest score (0.61), thanks to the inherent safety of OleA. However, a caveat is that relying solely on the hazard information listed in the SDS of each precursor overlooks a comprehensive toxicological evaluation of the HES itself, which may have a different profile.^24,75^ In some cases, mixtures exhibit toxicity despite being composed of individually nontoxic substances, or conversely, the toxicity of a precursor may depend on its partner in the eutectic mixture. For instance, in HES composed of Men and long-chain carboxylic acids, toxicity toward a keratinocyte cell line decreased with increasing chain length (C12–C18), with Men:DodA showing toxicity comparable to Men alone, while Men:stearic acid and Men:OleA displayed the lowest toxicity.^76,77^ Theoretical studies further indicate that both menthol and carboxylic acids can interact with cell membranes components. For instance, Men:OctA and OctA:DodA mixtures disrupt dipalmitoylphosphatidylcholine lipid bilayers, with the strongest effect observed for Men:OctA.^69^ Although a full investigation of HES safety is beyond the scope of the present work, a case-by-case evaluation remains essential to assess risks for both human health and ecosystems. Finally, economic sustainability has to be introduced as an important factor that can jeopardize the scalability of the process. When looking at the prices of the pure compounds, Men:OleA would have the highest production price among the four tested HES, while the all-fatty acid HES OctA:DodA would be the cheapest.

### Boosting the Sustainability: Evaluation of Reusability and Regeneration

The sections above demonstrate that the proposed HES are simple, effective, and promising candidates for water remediation of BPA and other contaminants. The sustainability assessment highlights, however, the need for process scale-up, aiming at waste reduction and increased throughput—ideally by incorporating automation. To move in this direction, scale-up tests with solvent reuse were performed using the model system TOPO:Men to evaluate the extraction performance over multiple extraction cycles. After each phase separation, the HES-rich phase as such was put in contact with fresh BPA-contaminated water for another extraction cycle, and the procedure was repeated 10 cycles (Figure 6a). Surprisingly, no reduction in extraction efficiency was observed up to the tenth cycle, with complete extraction achieved in all cycles. No detectable BPA signal appeared in the ^1^H NMR spectra of the water phase, indicating an EE consistently above 98.5% across all cycles (LOD = 1.56 ppm). This demonstrates that HES can be reused multiple times without compromising BPA extraction efficiency, even in the presence of BPA accumulation in the HES phase, further reinforcing their strong potential for contaminant removal. qNMR spectrum of the HES phase after the 10 cycles confirmed the presence of BPA at a concentration of 1000 ppm (Figure 6b).

**Figure 6:**
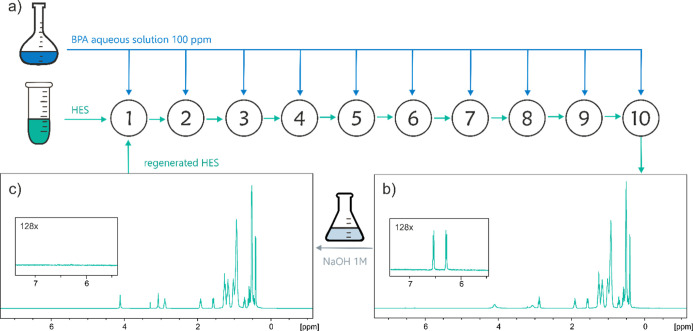
(a) Reuse protocol applied for TOPO:Men; (b) ^1^H NMR spectrum of the HES phase after the ten extraction cycles; and (c) ^1^H NMR spectrum of the HES phase after contact with NaOH 1 M solution.

Adapting an already published protocol,^31^ the BPA-enriched TOPO:Men phase after the tenth cycle was regenerated by back-extracting BPA with an equal volume of 1 M NaOH solution. NMR spectra of the two phases after separation proved the migration of the BPA phenolates to the water phase, as well as the chemical stability of the TOPO:Men after contact with the basic solution (Figure 6c). The regenerated TOPO:Men was used again to extract BPA, preserving an EE greater than 98.5% (via NMR quantification of the water phase). Given 1 volume of HES can treat up to 20 volumes of BPA-contaminated water, be regenerated with 1 volume of basic solution, and reused for further extraction, the process is promising and could be further optimized to enhance volume gain. Introducing the reuse and recycling steps strengthens the sustainability of the whole process, translating in highest scores when the Analytical EcoScale and the AGREEprep metrics are applied. As detailed in Table S14, when applying the Analytical EcoScale, less PPs are assigned to the “Waste” section (1 PP vs 6 PPs), with a final score of 95 vs 90 (Table 2). The same applies to the AGREEprep metrics: even though a higher number of steps have to be considered (criterion 7), recycling reduces waste (criterion 4) and offsets the use of less-than-fully sustainable solvents by allowing their reuse over multiple cycles (criterion 3) (Figures 5a,e and S21a,e).

## Conclusions

The HES evaluated in this study offer a compelling, ecofriendly alternative to conventional solvents for the removal of BPA from water. All proposed HES are water-immiscible, eliminating the need for salting-out agents and simplifying downstream separation. Our scouting criteria prioritized optimizing solvent handling properties and minimizing cross-contamination due to water-to-HES and HES-to-water leaching. The latter is a crucial point in developing sustainable extraction protocols, deserving a thorough and systematic investigation, to which this work gave one of the first extensive contribution.

Among the tested systems, TOPO:menthol stood out for its high extraction efficiency, minimal leaching, and operational robustness. Its performance remained consistent over ten reuse cycles, and it was successfully regenerated via back-extraction, underscoring the potential of HES-based processes to meet both environmental and industrial demands. While systematic studies of the HES-contaminant affinities across different chemical functionalities are still needed, the methodological approach developed here is broadly generalizable.

By combining efficiency, safety, and reusability, this work positions HES as promising candidates in the transition toward greener water treatment technologies while emphasizing the importance of a rational selection strategy that adequately accounts for cross-contamination. Although the efficacy of HES-based treatments still requires optimization under close-to-real conditions to compete with established LLE-based methods, their superior solvent sustainability and unparalleled potential for tunable, tailored solutions make them a highly compelling alternative.

## Supplementary Material



## Notes

Tables listing experimental results; UV–vis absorption spectra and calibration curves; ^1^H NMR spectra of all samples; tables listing LLE results; and green metrics analysis (PDF)

## §

INSTM and Chemistry for Technologies Laboratory, Department of Mechanical and Industrial Engineering, University of Brescia, Via Branze 38, 25123 Brescia, Italy
