# Supplementary material for: Hydrophobic Eutectic Solvents for Sustainable Bisphenol A Extraction from Water: Screening and Selection Based on Key Performance Criteria
Source: ACS Omega. 2025 Sep 16;10(38):44662–74. doi: 10.1021/acsomega.5c07390 (PMC12489678; doi:10.1021/acsomega.5c07390)
Supplement: Supplementary file 1 [file ao5c07390_si_001.pdf]

Supporting Information to

Hydrophobic Eutectic Solvents for Sustainable Bisphenol A Extraction from Water: Screening and Selection Based on Key Performance Criteria

*Chiara Carotti,[a] Grazia Isa C. Righetti,[a] Alberto Mannu,[a†] Arianna Rossetti,[a,b] Laura Riva,[a] Gloria Nicastro,[a] Francesco Briatico Vangosa,[a] Carlo Punta,[a] Andrea Mele,[a] and Maria Enrica Di Pietro[a]**

[a] Department of Chemistry, Materials and Chemical Engineering "Giulio Natta", Politecnico di Milano, Piazza Leonardo da Vinci 32, 20133, Milano, Italy

[b] INSTM—Local Unit c/o Politecnico di Milano, Via Mancinelli 7, Milano, 20131, Italy

† Present address: INSTM and Chemistry for Technologies Laboratory, Department of Mechanical and Industrial Engineering, University of Brescia, Via Branze 38, 25123 Brescia, Italy

Corresponding Author: mariaenrica.dipietro@polimi.it

Experimental section

Table S1. List of compounds used in this work. The compounds were used as received without further purification.

Compound	Abbreviation	Supplier	CAS number	Purity [a]
DL-Menthol	Men	Sigma-Aldrich	89-78-1	≥ 98 %
Thymol	Thy	Sigma-Aldrich	89-83-8	≥ 98.5 %
(R)-(+)-Camphor	Cam	Sigma-Aldrich	464-49-3	≥ 98 %
(R)-(-)-Carvone	Car	Sigma-Aldrich	6485-40-1	≥ 97 %
Hexanoic acid, Caproic acid	HexA	Sigma-Aldrich	142-62-1	≥ 99 %
Octanoic Acid, Caprylic Acid	OctA	Sigma-Aldrich	124-07-2	≥ 99 %
Decanoic acid, Capric acid	DecA	Sigma-Aldrich	334-48-5	≥ 98 %
Lauric Acid, Dodecanoic Acid	DodA	Sigma-Aldrich	143-07-7	≥ 99 %
Oleic Acid, cis-9-Octadecenoic acid	OleA	Sigma-Aldrich	112-80-1	≥ 99 %
Trioctylphosphineoxide	TOPO	TCI chemicals	78-50-2	>95%
Deuterated water	D_2O	Sigma-Aldrich	7789-20-0	99.9 atom % D
Dimethyl sulfoxide-d_6	DMSO-d_6	Sigma-Aldrich	2206-27-1	99.9 atom % D
3-(Trimethylsilyl)propionic-2,2,3,3-d_4 acid sodium salt	TSP	Isotec	24493-21-8	98%
Tetramethylsilane	TMS	Sigma-Aldrich	75-76-3	≥ 99.0 %

[a] As stated by the supplier.

Table S2. Density and viscosity of freshly prepared HES and viscosity after one month.

HES	density (kg m^{-3})	viscosity (mPa s)	viscosity after one month (mPa s)
Men:Cam	933.9±3.8	21.17±0.09	21.37±0.01
Thy:Cam	981.3±1.5	20.84±0.02	20.95±0.00
Men:HexA	928.7±10.9	9.84±0.03	9.97±0.01
Men:OctA	925.9±11.1	12.71±0.18	12.54±0.01
Men:DecA	903.1±9.6	21.82±0.14	21.56±0.04
Men:OleA	904.4±5.5	31.34±0.01	32.13±0.18
TOPO:Men	915.7±8.9	41.47±0.94	41.11±0.18
TOPO:DodA	911.6±3.9	50.16±0.20	51.65±0.18
Car:Men	948.2±6.8	3.34±0.10	3.31±0.01
Car:OctA	968.1±7.2	3.03±0.01	3.10±0.01
Car:DecA	968.4±9.8	3.23±0.05	3.37±0.03
OctA:DecA	910.5±6.9	6.30±0.02	6.38±0.00
OctA:DodA	924.8±3.9	7.29±0.05	7.47±0.04

Table S3. HES leaching after mixing with water.

HES	Component 1 (wt%)		Component 2 (wt%)
Men:Cam	0.036±0.007		0.193±0.011
Thy:Cam	0.067±0.002		0.124±0.010
Men:HexA	0.027±0.003		1.115±0.003
Men:OctA	0.041±0.005		0.093±0.005
Men:DecA	0.048±0.004		0.017±0.005
Men:OleA	0.049±0.005		0.015±0.015
TOPO:Men	/		0.044±0.003
TOPO:DodA[a]		0.003±0.001	
Car:Men	0.134±0.005		0.063±0.006
Car:OctA	0.133±0.003		0.131±0.09
Car:DecA	0.126±0.005		0.016±0.005
OctA:DecA[a]		0.155±0.018	
OctA:DodA[a]		0.065±0.005	

[a] only the average leaching is available

Table S4. Solubility of HES components in water at 25°C (unless otherwise indicated).

Precursor	Solubility (mg/L)
Men	456 [a]
Thy	800 [b]
Cam	1600 [c]
Car	1310 [d]
HexA	10300 [e]
OctA	680 *[f]
DecA	61.8 [g]
DodA	4.81 [h]
OleA	none [i]
TOPO	none [j]

[a] https://www.sigmaaldrich.com/IT/en/sds/sial/m2772?userType=anonymous

[b] https://www.sigmaaldrich.com/IT/en/sds/sigma/t0501?userType=anonymous

[c] https://pubchem.ncbi.nlm.nih.gov/compound/Camphor#section=Solubility

[d] https://pubchem.ncbi.nlm.nih.gov/compound/439570#section=Boiling-Point

[e] https://www.sigmaaldrich.com/IT/en/sds/aldrich/153745?userType=anonymous

[f] https://www.sigmaaldrich.com/IT/en/sds/sigma/c2875?userType=anonymous

* at 20°C

[g] https://www.sigmaaldrich.com/IT/en/sds/sigma/c1875?userType=anonymous

[h] https://pubchem.ncbi.nlm.nih.gov/compound/3893#section=Solubility

[i] https://pubchem.ncbi.nlm.nih.gov/compound/445639#section=Solubility

[j] https://datasheets.scbt.com/sc-216016.pdf

Table S5. pH change (ΔpH) of the water phase after contact with HES, and water content of neat and water-saturated HES.

HES	ΔpH	water content of neat HES (wt%)	water content of saturated HES (wt%)
Men:Cam	0.19	0.158±0.004	2.520±0.005
Thy:Cam	0.84	0.120±0.013	2.211±0.002
Men:HexA	2.96	0.173±0.004	3.134±0.001
Men:OctA	2.09	0.199±0.016	2.843±0.002
Men:DecA	1.24	0.178±0.011	2.542±0.002
Men:OleA	1.57	0.073±0.009	1.320±0.001
TOPO:Men	0.15	0.229±0.027	2.598±0.001
TOPO:DodA	0.19	0.224±0.008	1.818±0.001
Car:Men	1.26	0.080±0.011	1.543±0.001
Car:OctA	1.94	0.084±0.006	2.183±0.001
Car:DecA	1.65	0.070±0.005	1.572±0.001
OctA:DecA	2.17	0.279±0.052	1.907±0.001
OctA:DodA	2.15	0.235±0.012	1.667±0.001

Table S6. EcoScale assessment for all HES prepared in this work.

	Parameters	Penalty points												
		Men:Cam 2:1	Thy:Cam 1:1	Men:HexA 1:1	Men:OctA 1:1	Men:DecA 2:1	Men:OleA 2:1	TOPO:Men 1:2	TOPO:DodA 1:1	Car:Men 3:1	Car:OctA 4:1	Car:DecA 9:1	OctA:DecA 3:1	OctA:DodA 3:1
1	Yield[a]	0	0	0	0	0	0	0	0	0	0	0	0	0
2	Price (for 10mmol of end product)[b]	0	0	0	0	0	3	0	0	0	0	0	0	0
3	Safety[c]	10	20	0	5	5	0	5	5	5	10	10	10	5
4	Setup[d]	0	0	0	0	0	0	0	0	0	0	0	0	0
5	Temperature/time[e]	2	2	2	2	2	2	2	2	2	2	2	2	2
6	Workup and purification[f]	0	0	0	0	0	0	0	0	0	0	0	0	0
	Total penalty points	12	22	2	7	7	5	7	7	7	12	12	12	7
	Ecoscale	88	78	98	93	93	95	93	93	93	88	88	88	93

[a] PPs = (100-%yield)/2

[b] PPs = 0 for inexpensive components (< $10) and PPs = 3 for expensive components (> $10 and < $50)

[c] PPs = 0 for Men, DodA, HexA, and OleA; PPs = 5 for OctA, DecA, and TOPO (N = dangerous for environment); PPs = 5 for Car (T = toxic); PPs = 10 for Thy (N = dangerous for environment + T = toxic); PPs = 10 for Cam (F = flammable + T = toxic)

[d] PPs = 0 for common setup

[e] PPs = 2 for heating, < 1h

[f] PPs = 0 for no workup and purification

Analytical methods: additional tables and figures

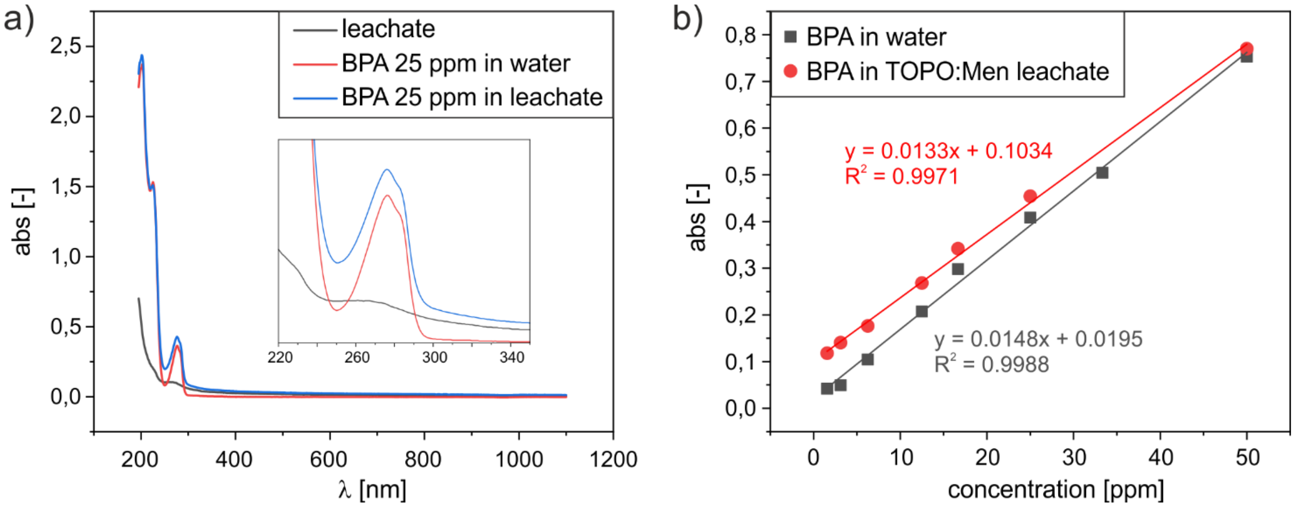

Figure S1. (a) UV-Vis spectra of a water phase after mixing with TOPO:Men (leachate, black line), and two water solutions of BPA 25 ppm without (BPA in water, red line) and with (BPA in leachate, blue line) HES leaching (a). (b) UV-Vis calibration curves for bisphenol A in water (dark grey squares) and water after mixing with TOPO:Men (red circles).

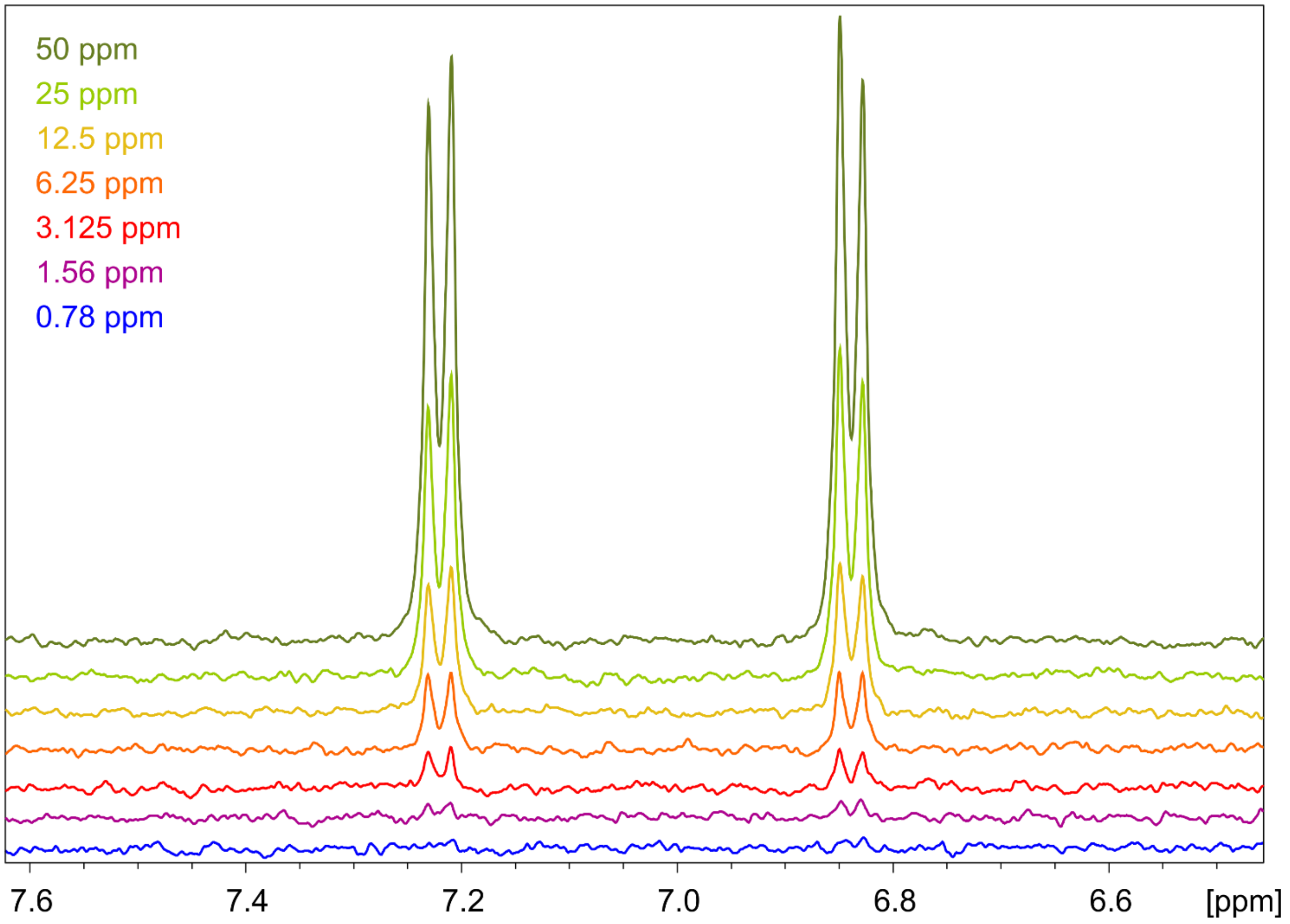

Figure S2. Enlargement of the 1D ^1H NMR spectra at 25 °C of water solution of BPA at increasing concentrations corresponding to the aromatic protons of BPA.

Table S7. Comparison between UV-Vis and NMR quantification methods used in the present LLE protocol.

Parameter	UV-Vis	NMR
Cost	low	high
Speed / Throughput	rapid	intermediate
Experimental time	short	intermediate
Detection limits	low	intermediate
Accessibility	wide	limited
Energy consumption	low	high
Sample preparation	none	minimal
Personnel training	low	intermediate
Sensitivity to matrix-effects	high	low
Leaching quantification	not directly measurable	directly quantifiable

Additional NMR spectra and data

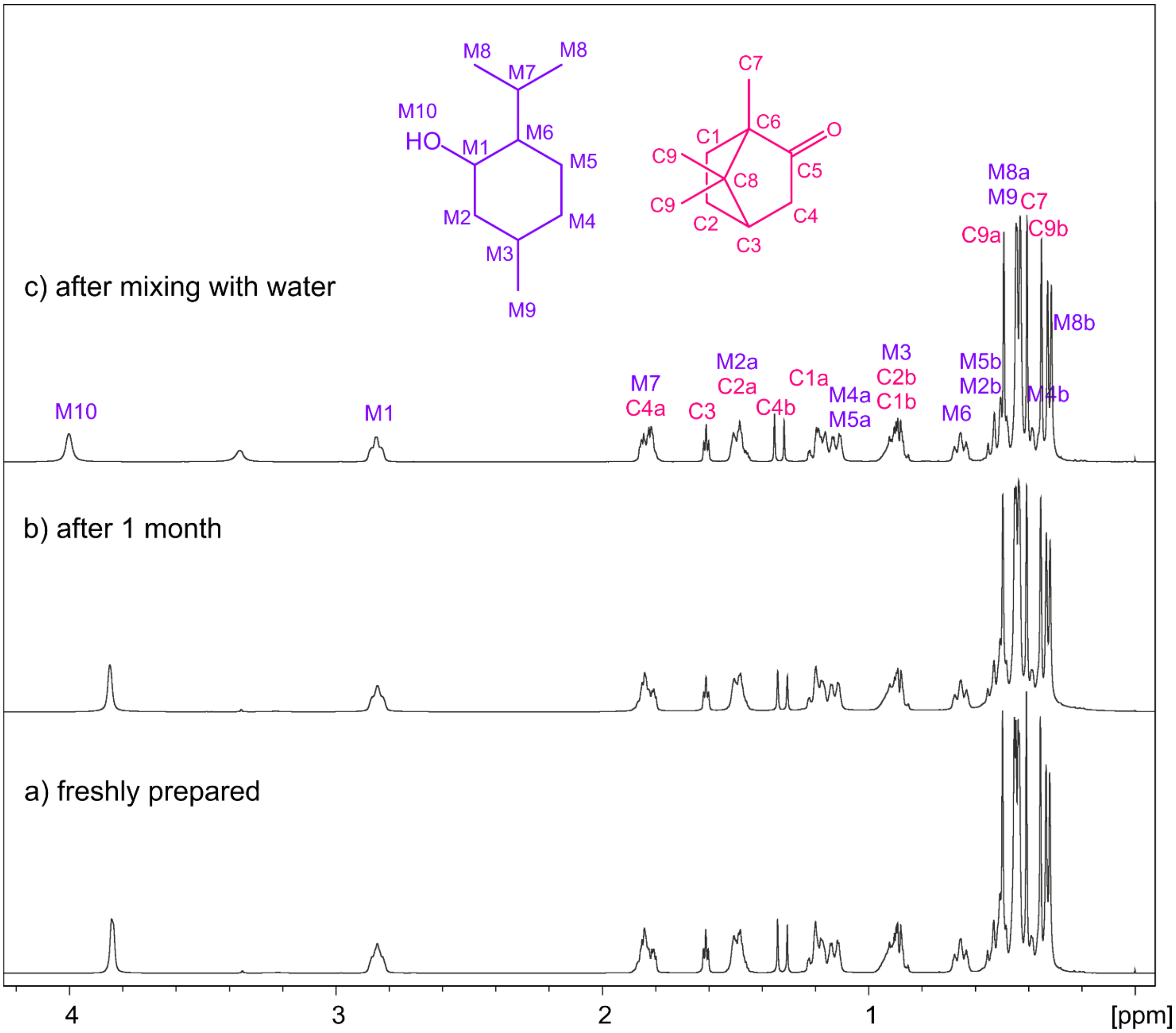

Figure S3. 1D ^{1}H NMR spectra at 25 °C of Men:Cam freshly prepared (a), one month after preparation (b), and after mixing with water (c).

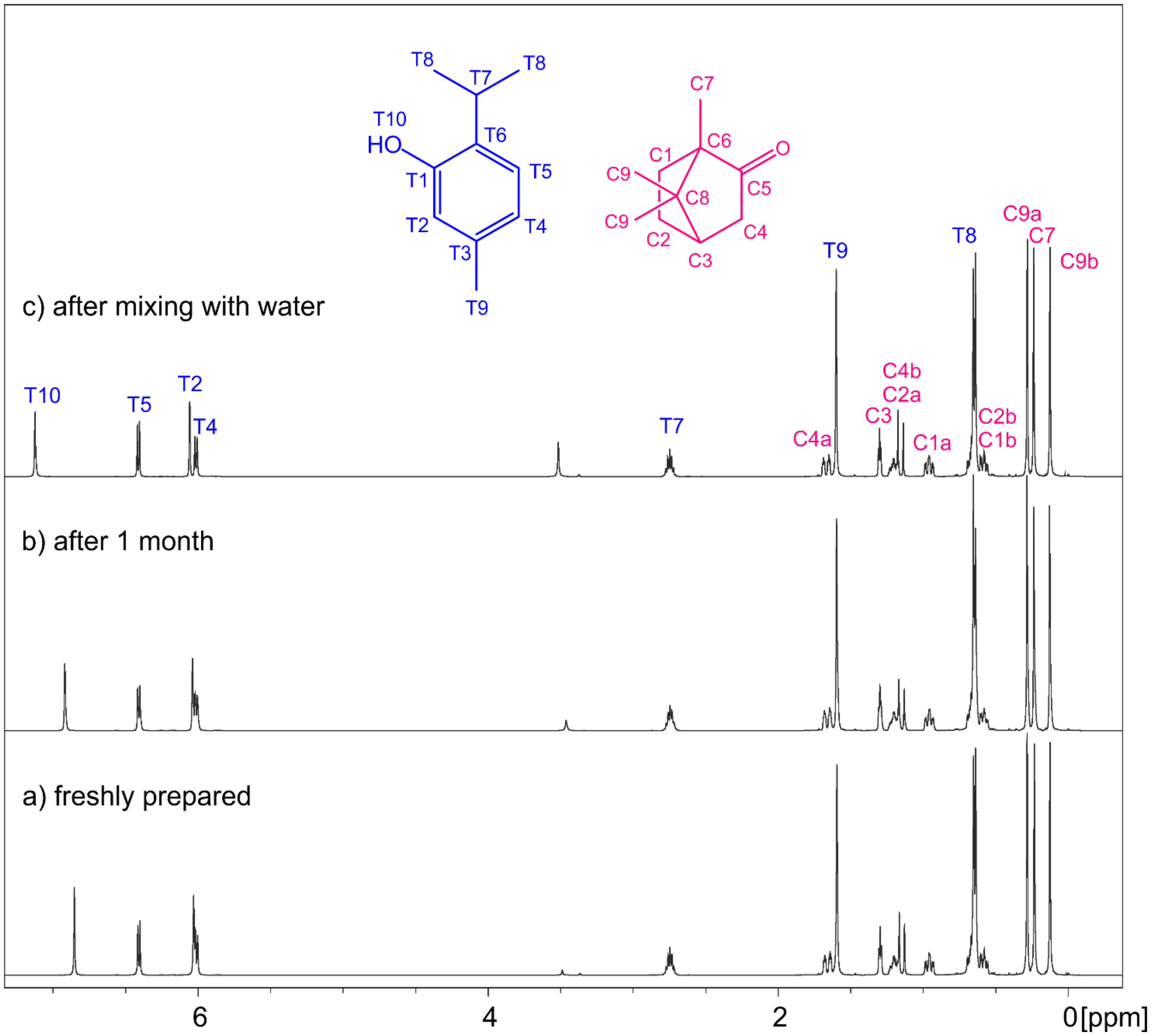

Figure S4. 1D ^{1}H NMR spectra at 25 °C of Thy:Cam freshly prepared (a), one month after preparation (b), and after mixing with water (c).

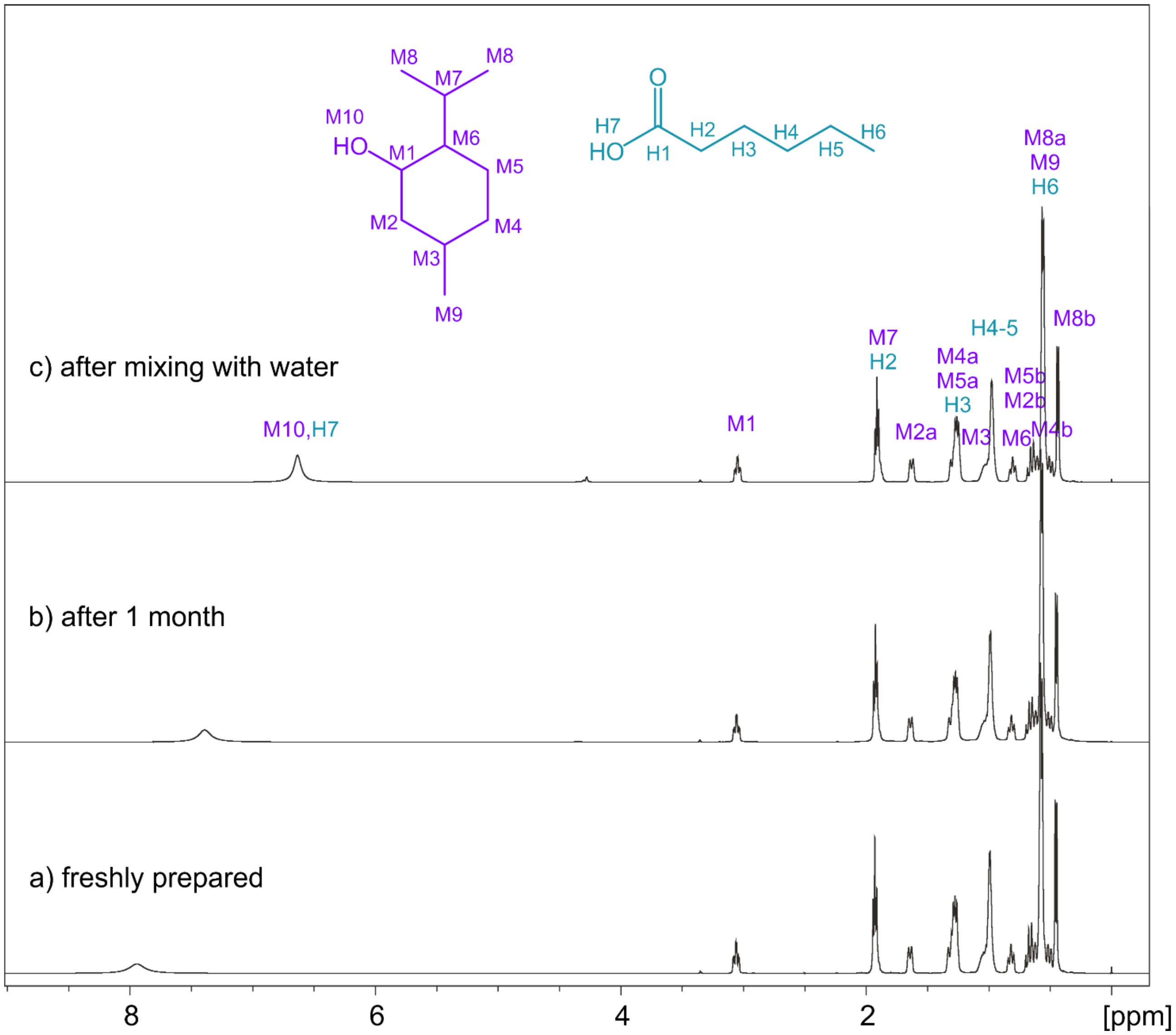

Figure S5. 1D ^{1}H NMR spectra at 25 °C of Men:HexA freshly prepared (a), one month after preparation (b), and after mixing with water (c).

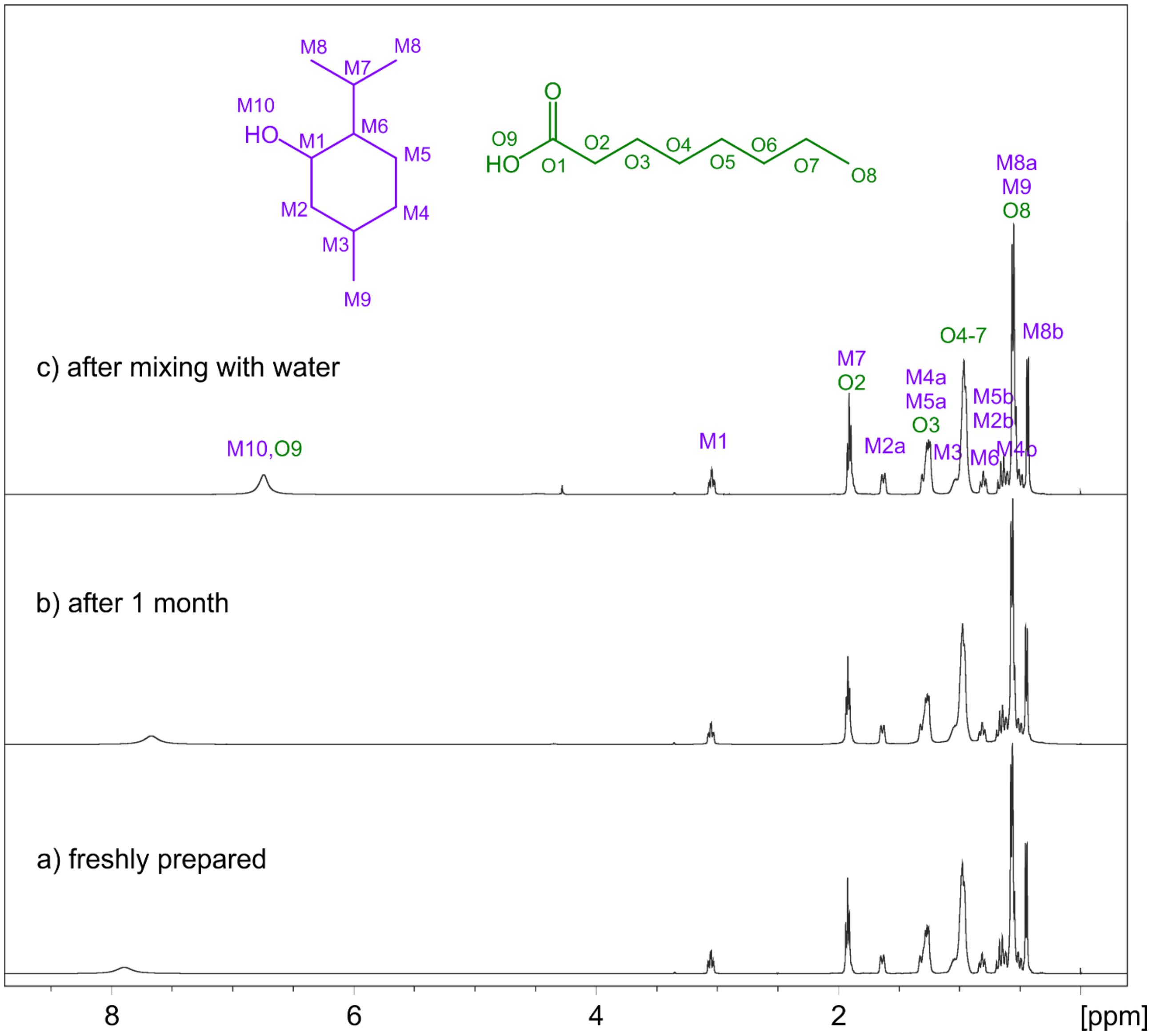

Figure S6. 1D ^{1}H NMR spectra at 25 °C of Men:OctA freshly prepared (a), one month after preparation (b), and after mixing with water (c).

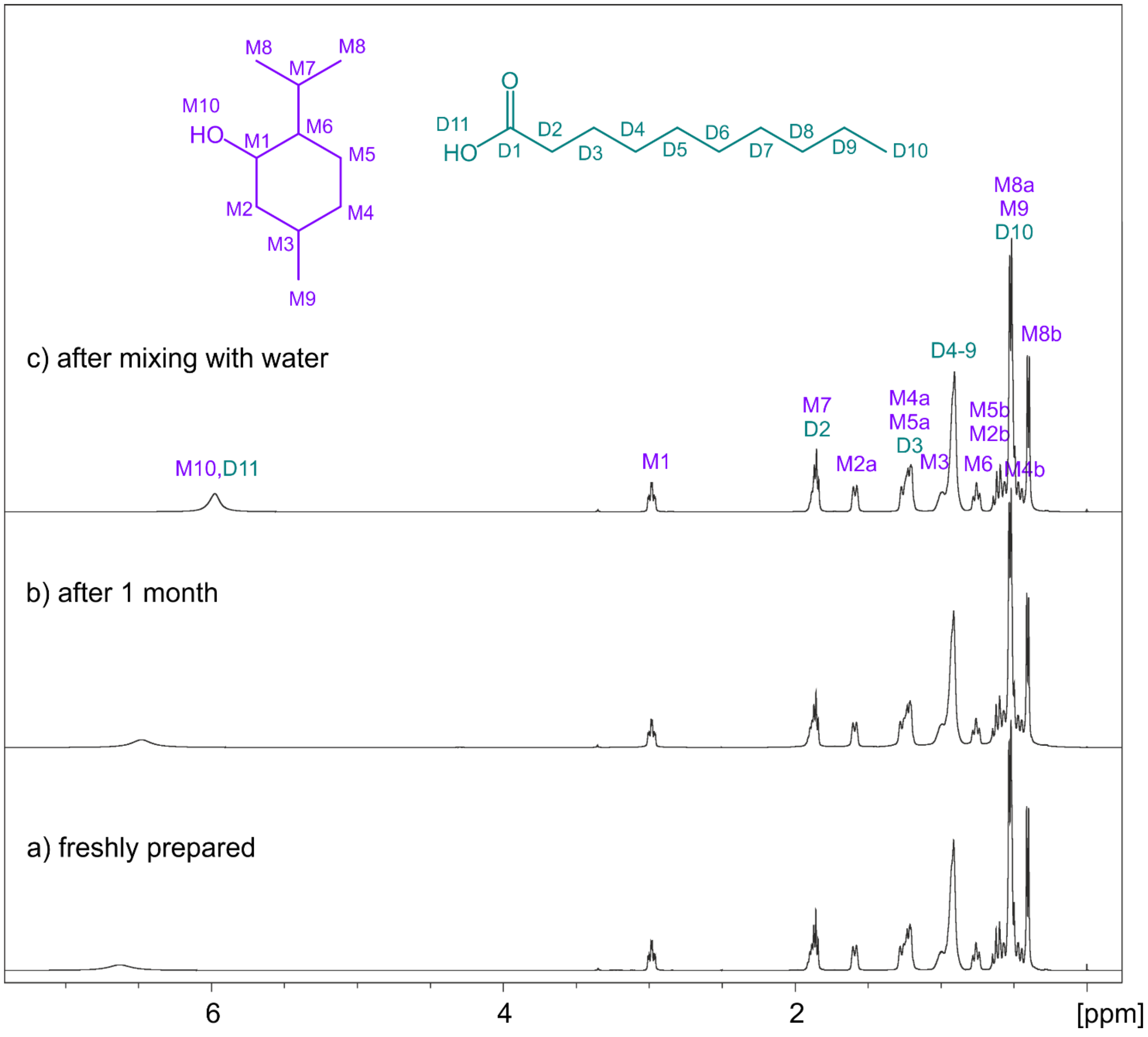

Figure S7. 1D ^{1}H NMR spectra at 25 °C of Men:DecA freshly prepared (a), one month after preparation (b), and after mixing with water (c).

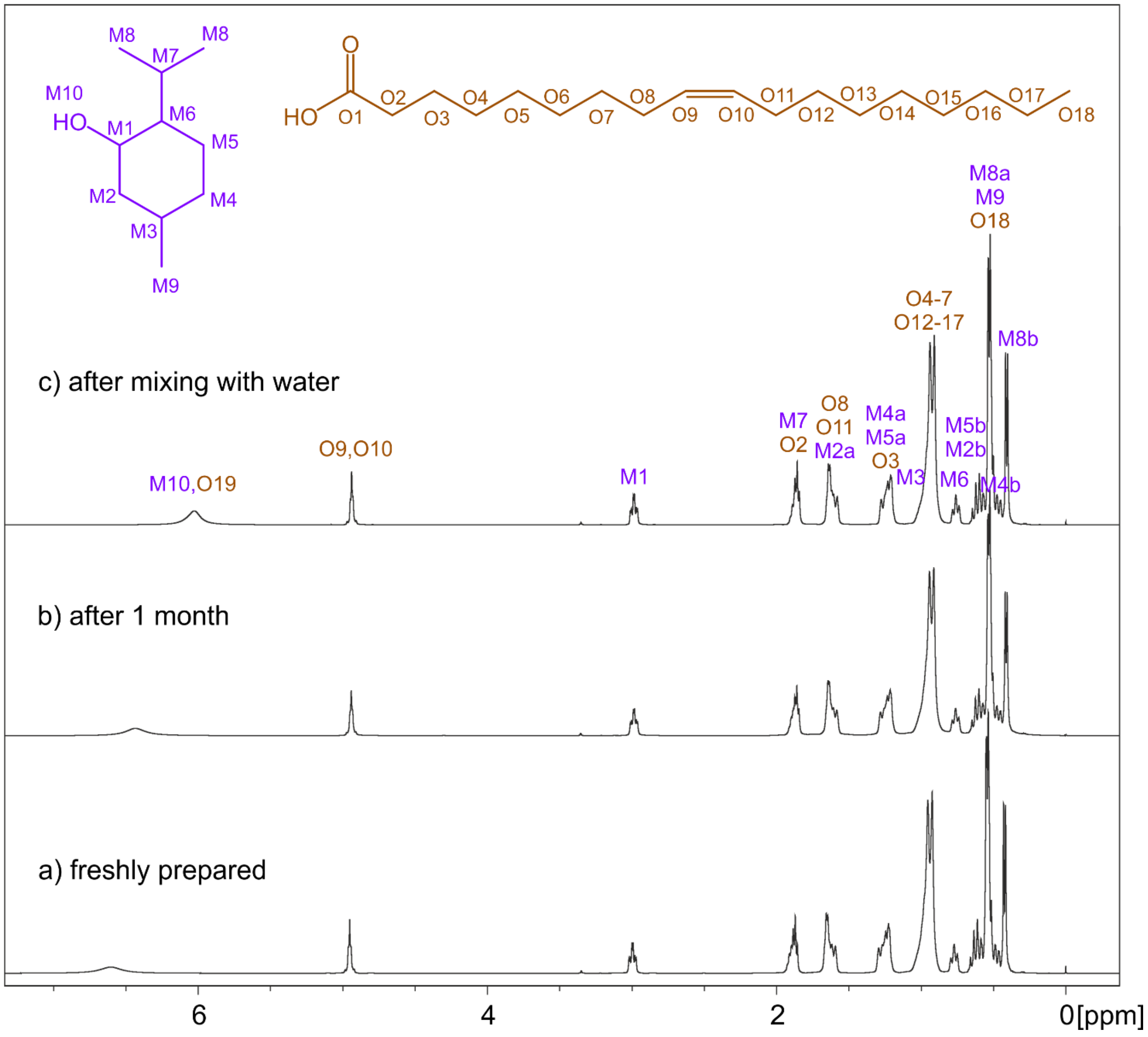

Figure S8. 1D ^{1}H NMR spectra at 25 °C of Men:OleA freshly prepared (a), one month after preparation (b), and after mixing with water (c).

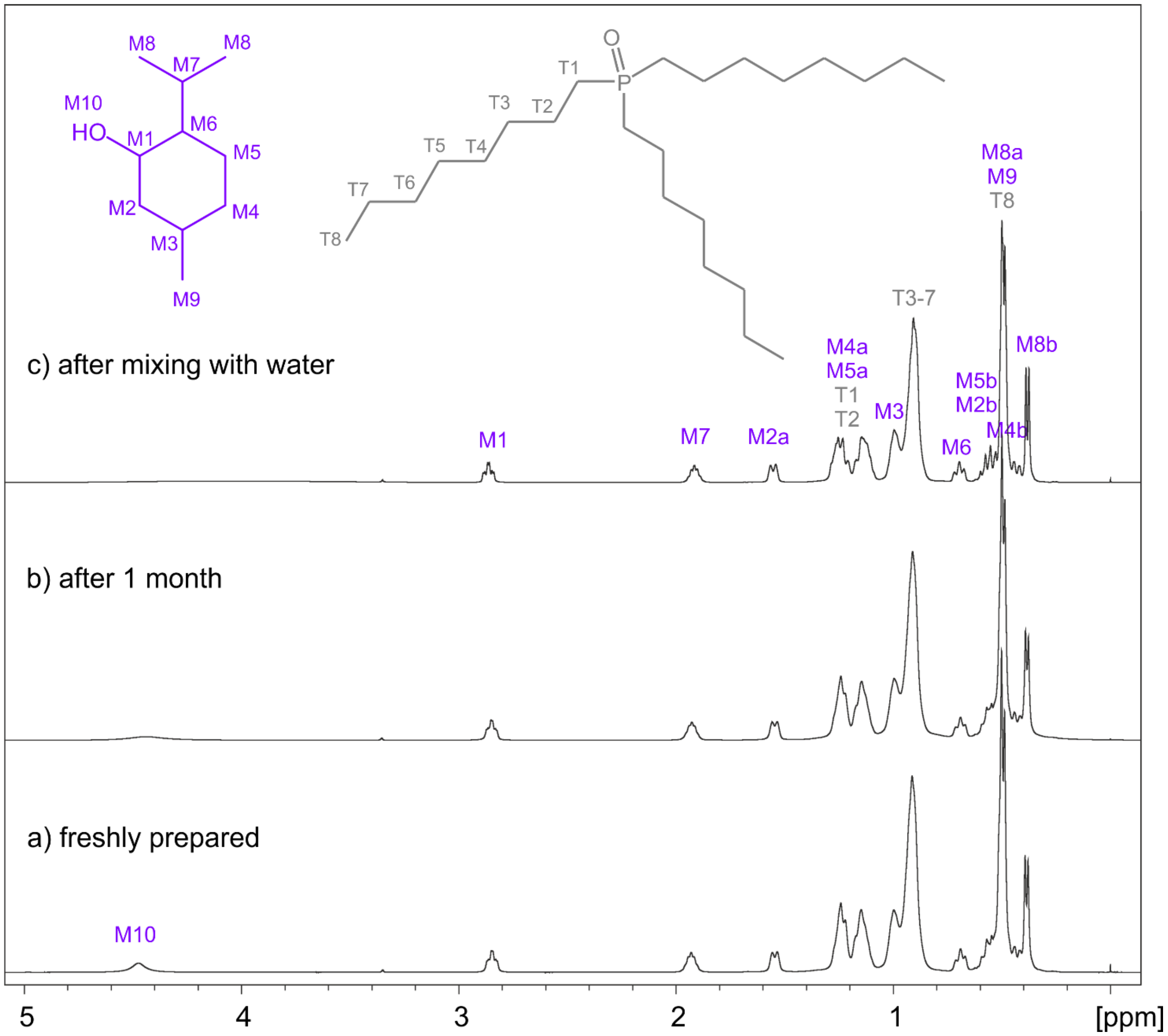

Figure S9. 1D ^{1}H NMR spectra at 25 °C of TOPO:Men freshly prepared (a), one month after preparation (b), and after mixing with water (c).

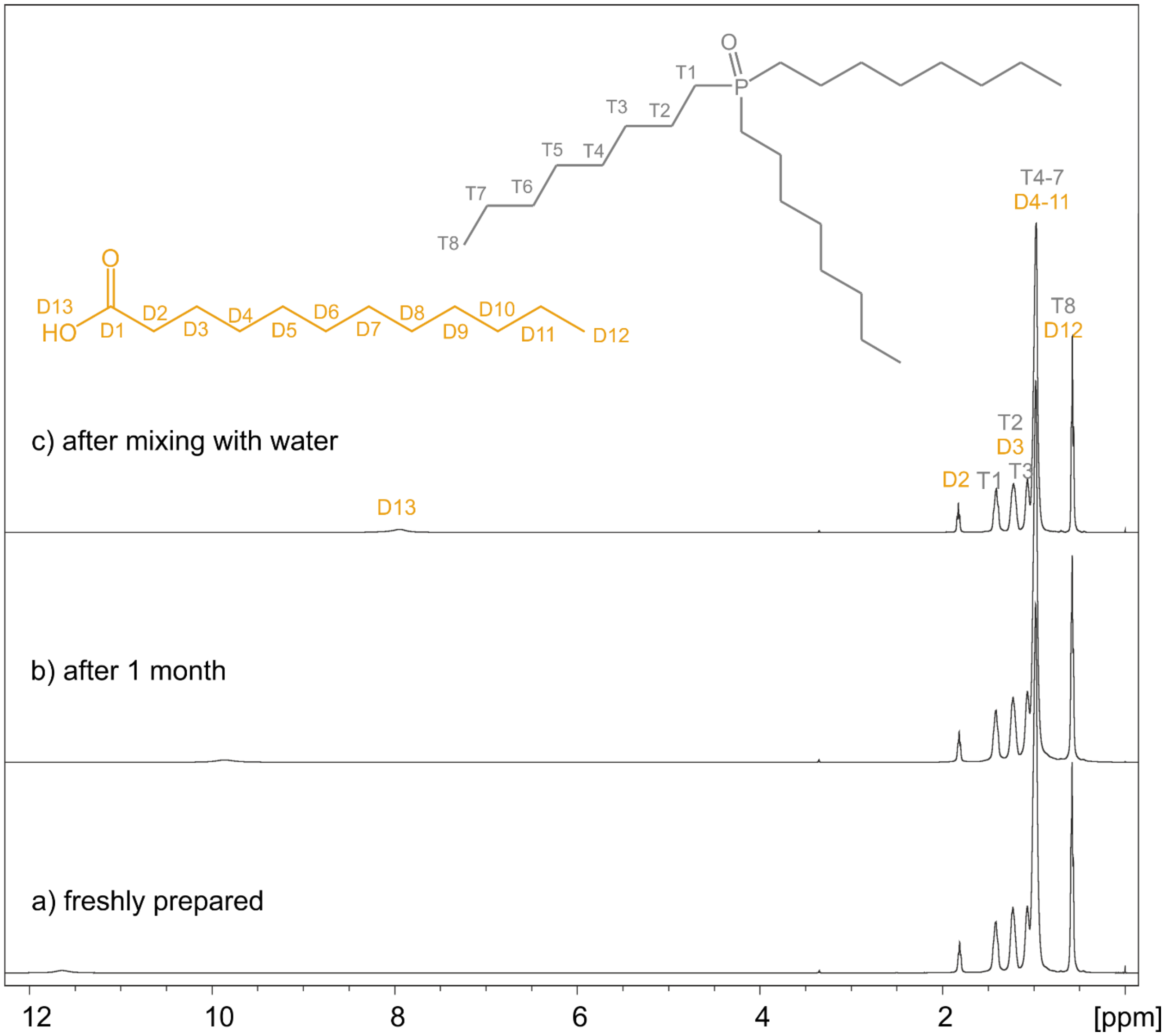

Figure S10. 1D ^{1}H NMR spectra at 25 °C of TOPO:DodA freshly prepared (a), one month after preparation (b), and after mixing with water (c).

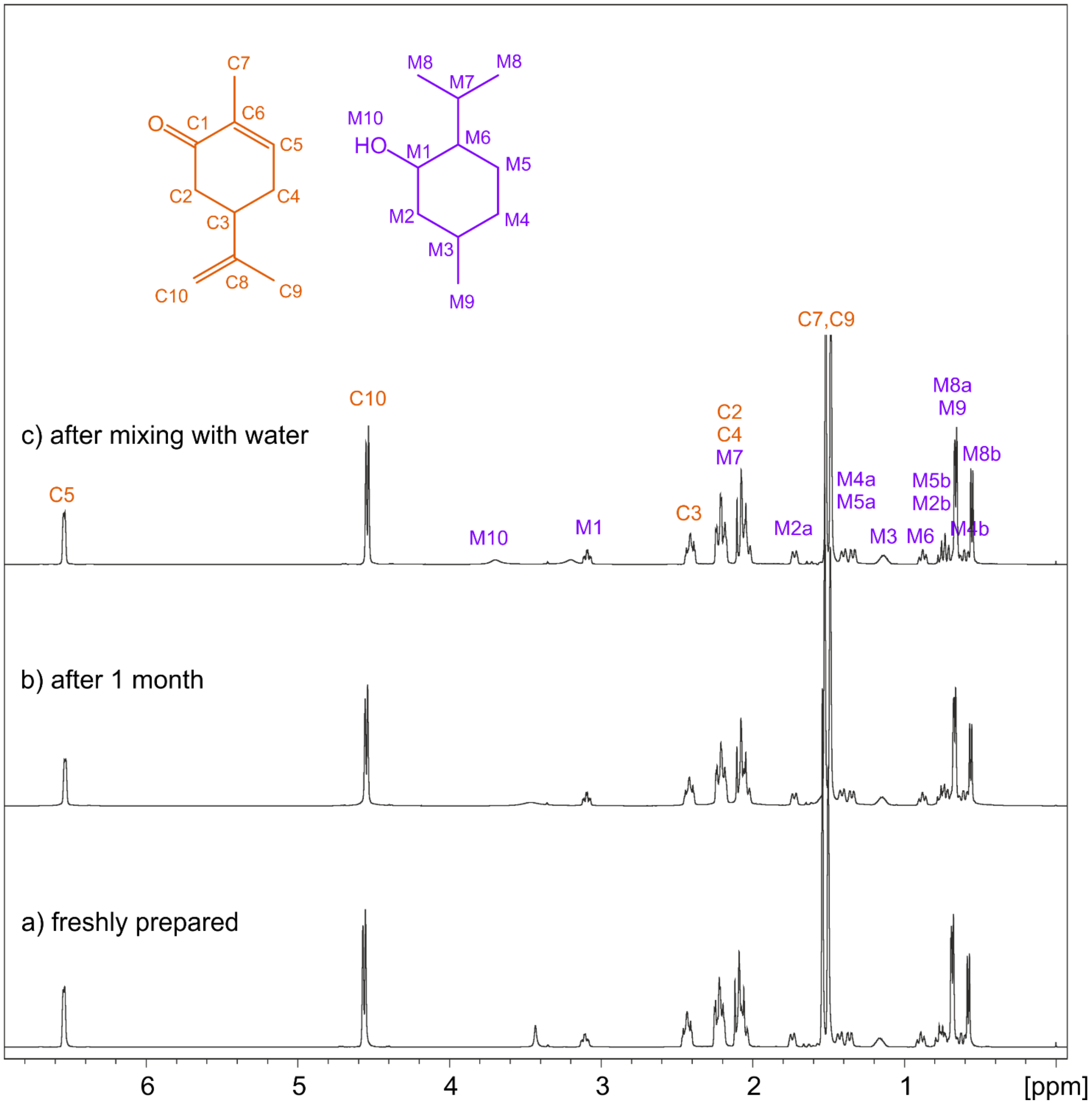

Figure S11. 1D ^{1}H NMR spectra at 25 °C of Car:Men freshly prepared (a), one month after preparation (b), and after mixing with water (c).

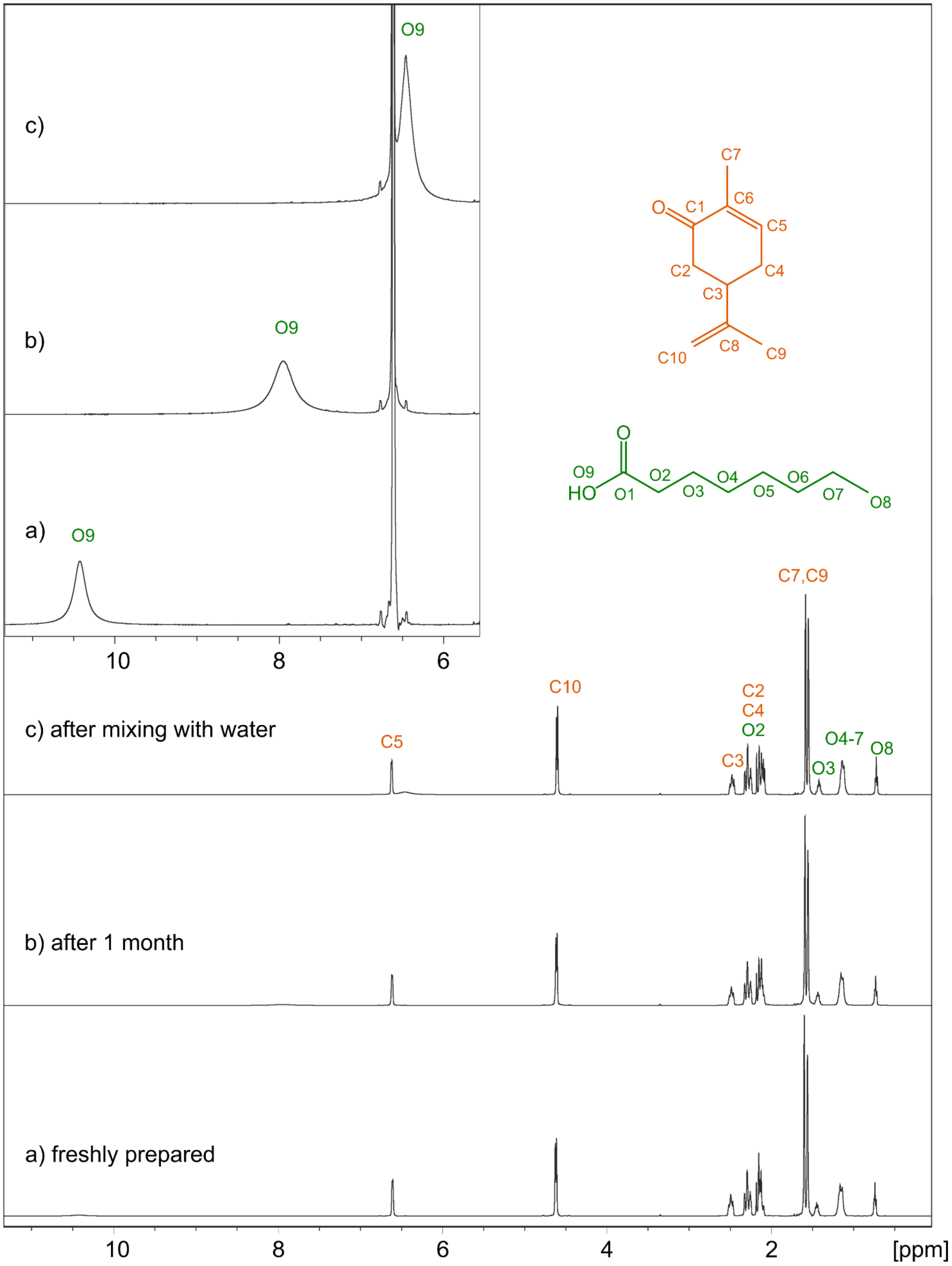

Figure S12. 1D ^{1}H NMR spectra at 25 °C of Car:OctA freshly prepared (a), one month after preparation (b), and after mixing with water (c).

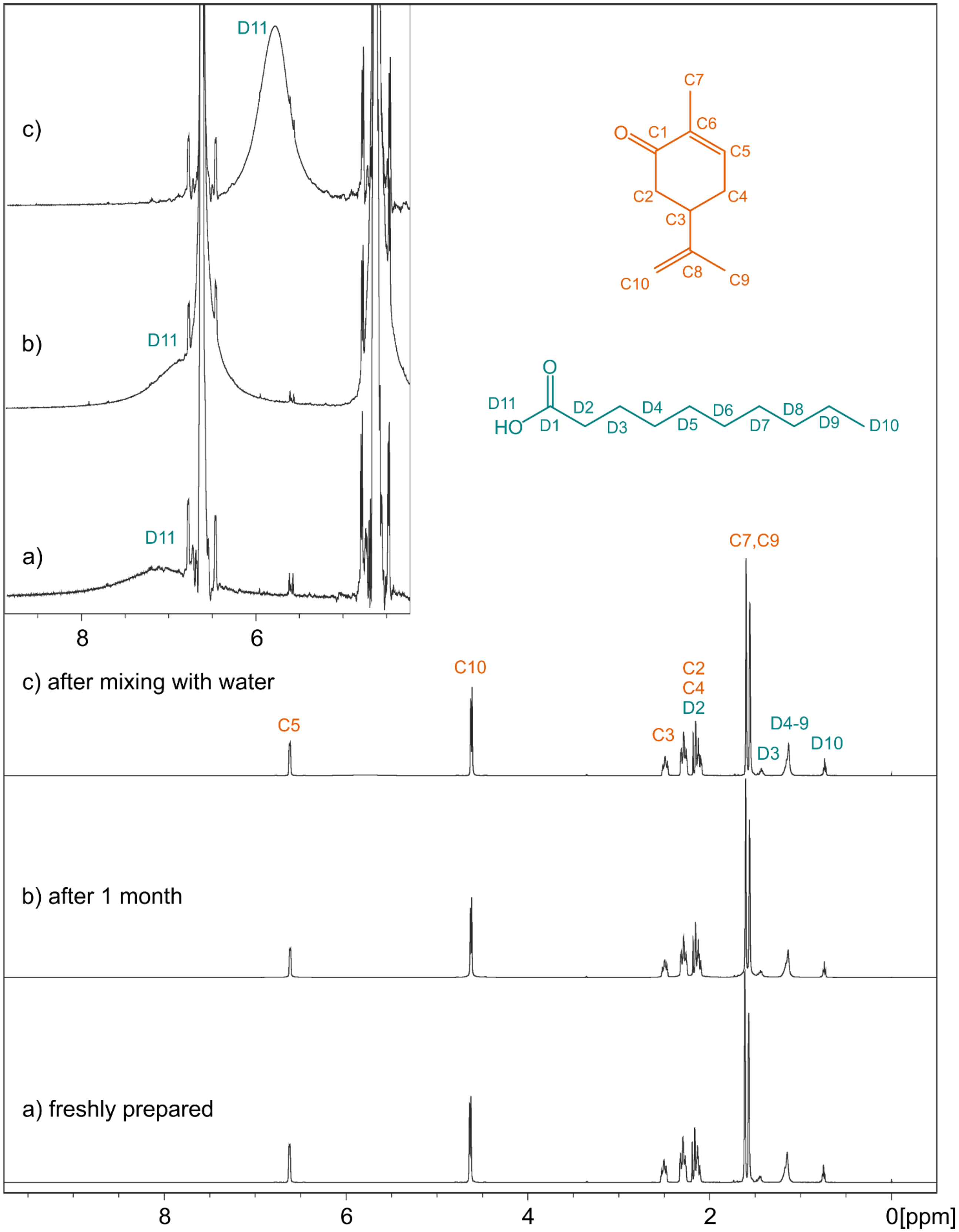

Figure S13. 1D ^{1}H NMR spectra at 25 °C of Car:DecA freshly prepared (a), one month after preparation (b), and after mixing with water (c).

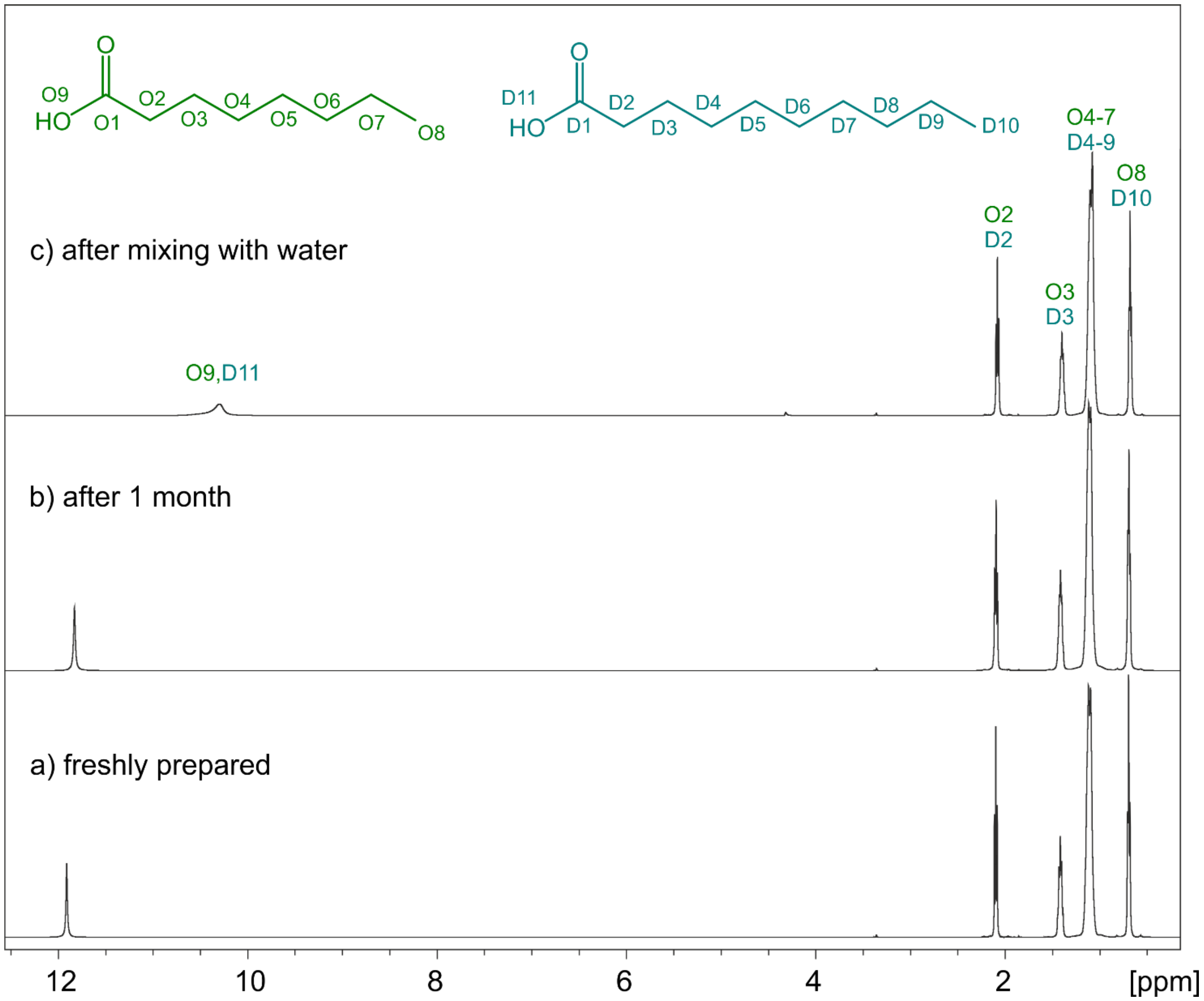

Figure S14. 1D ^{1}H NMR spectra at 25 °C of OctA:DecA freshly prepared (a), one month after preparation (b), and after mixing with water (c).

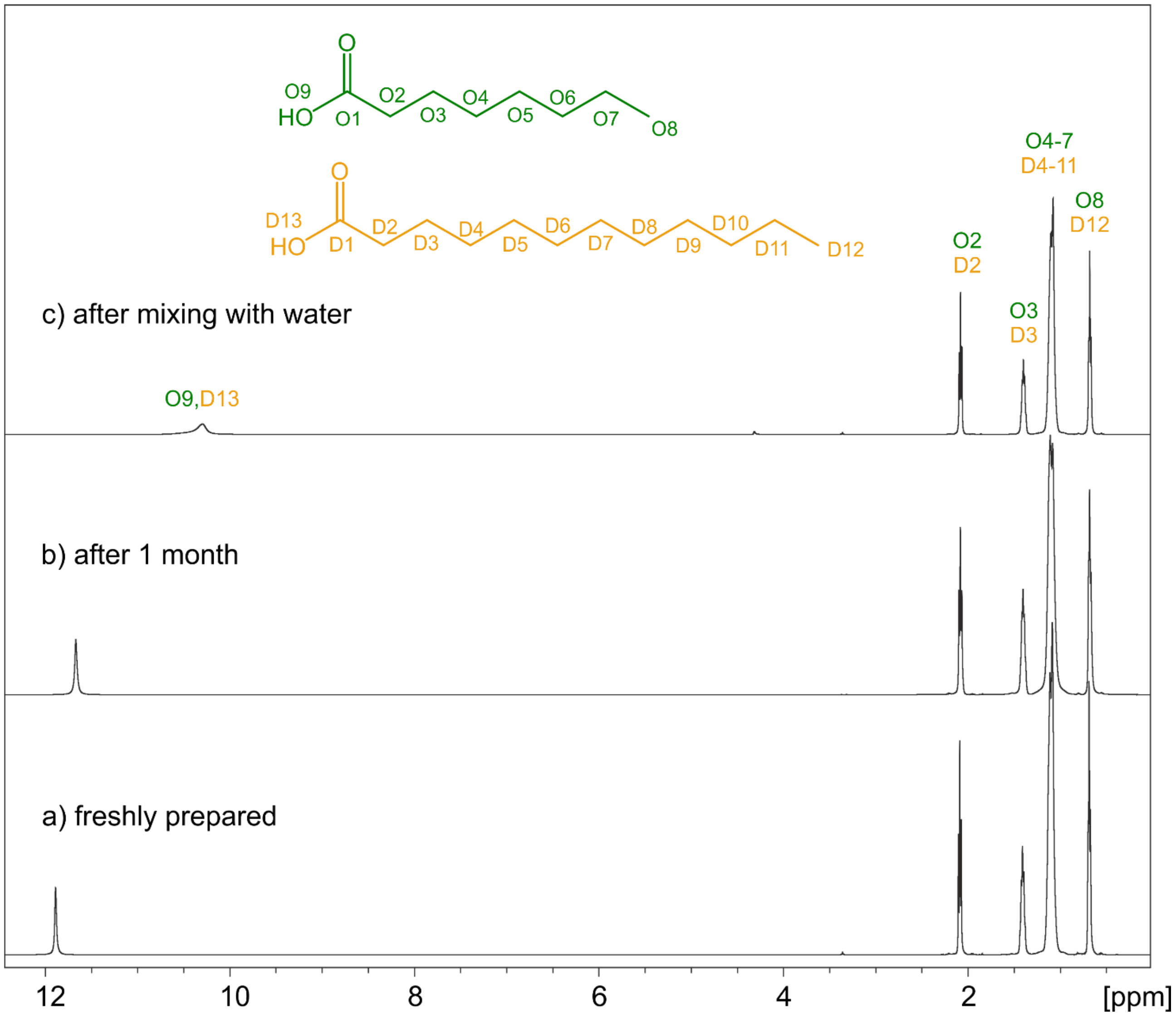

Figure S15. 1D ^1H NMR spectra at 25 °C of OctA:DodA freshly prepared (a), one month after preparation (b), and after mixing with water (c).

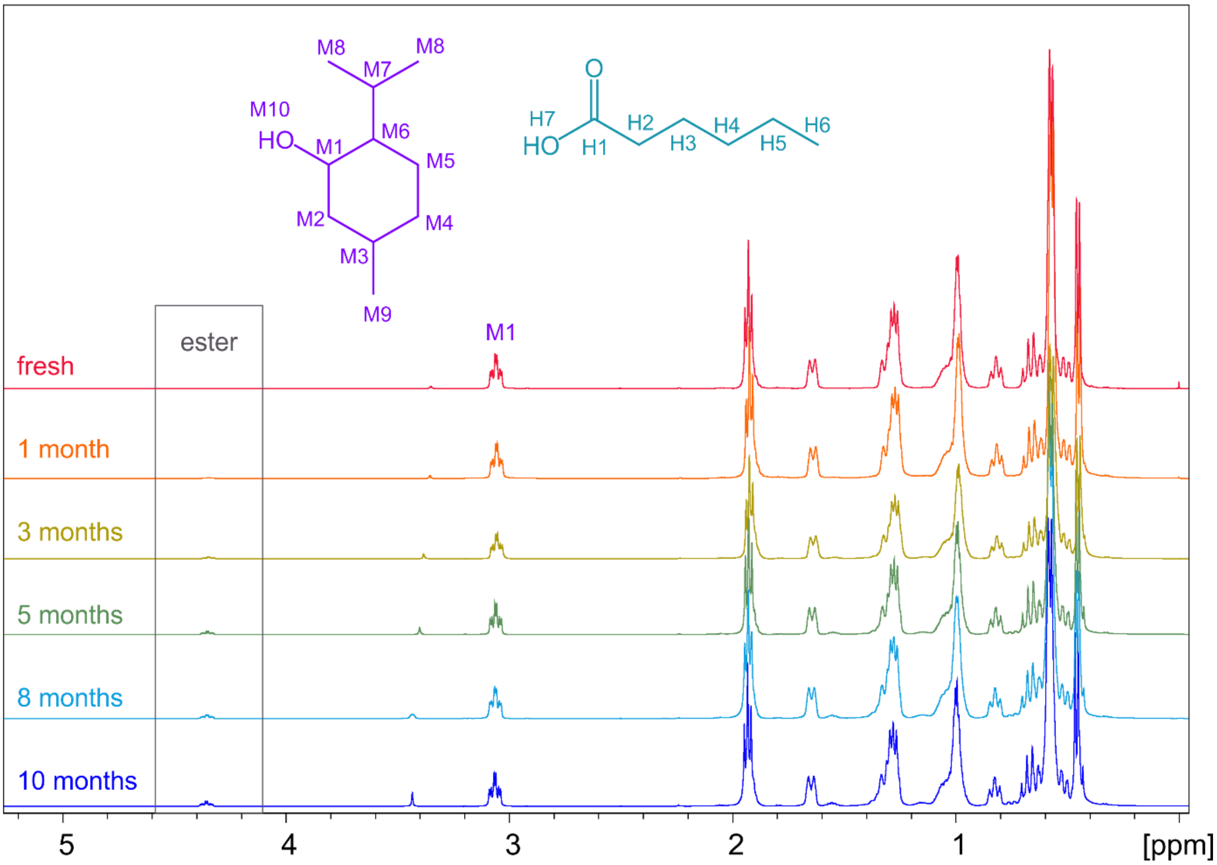

Figure S16. 1D ^{1}H NMR spectra at 25 °C of Men:HexA at different time after preparation.

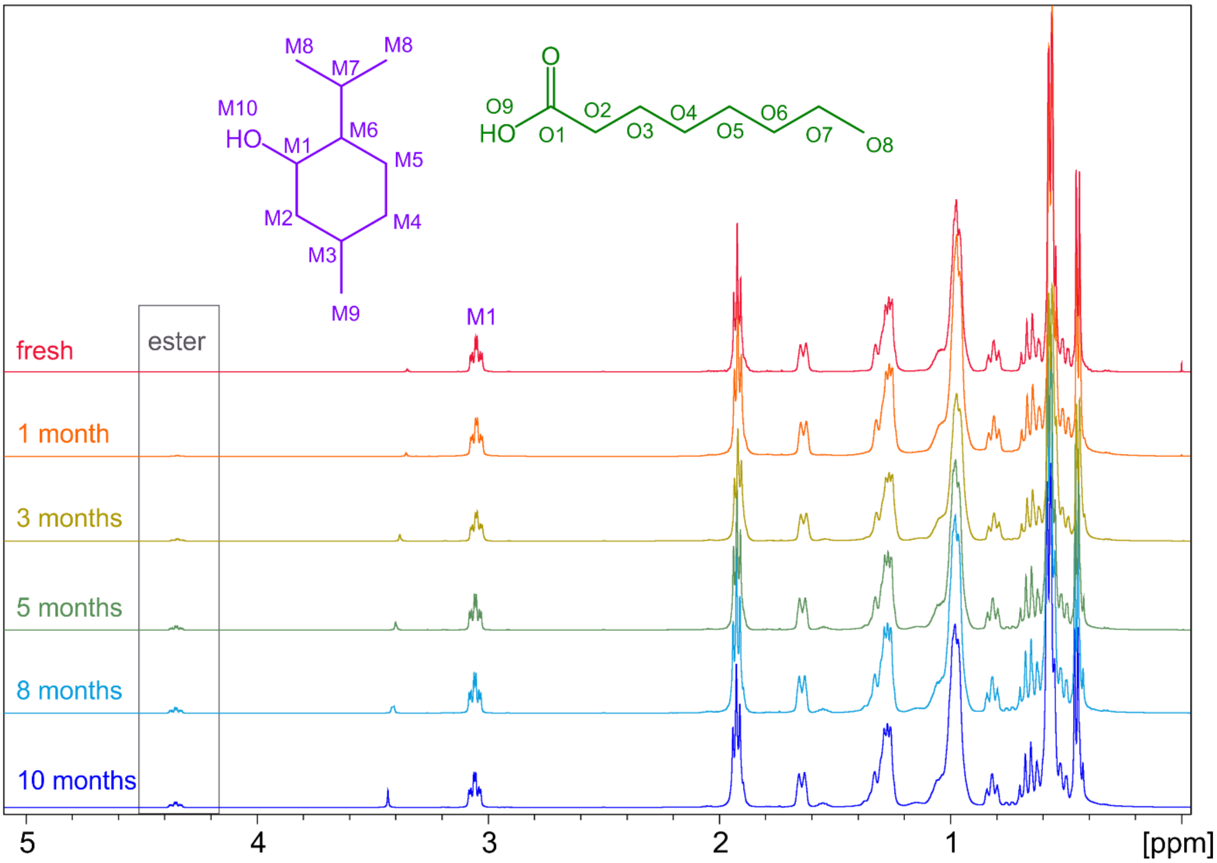

Figure S17. 1D ^{1}H NMR spectra at 25 °C of Men:OctA at different time after preparation.

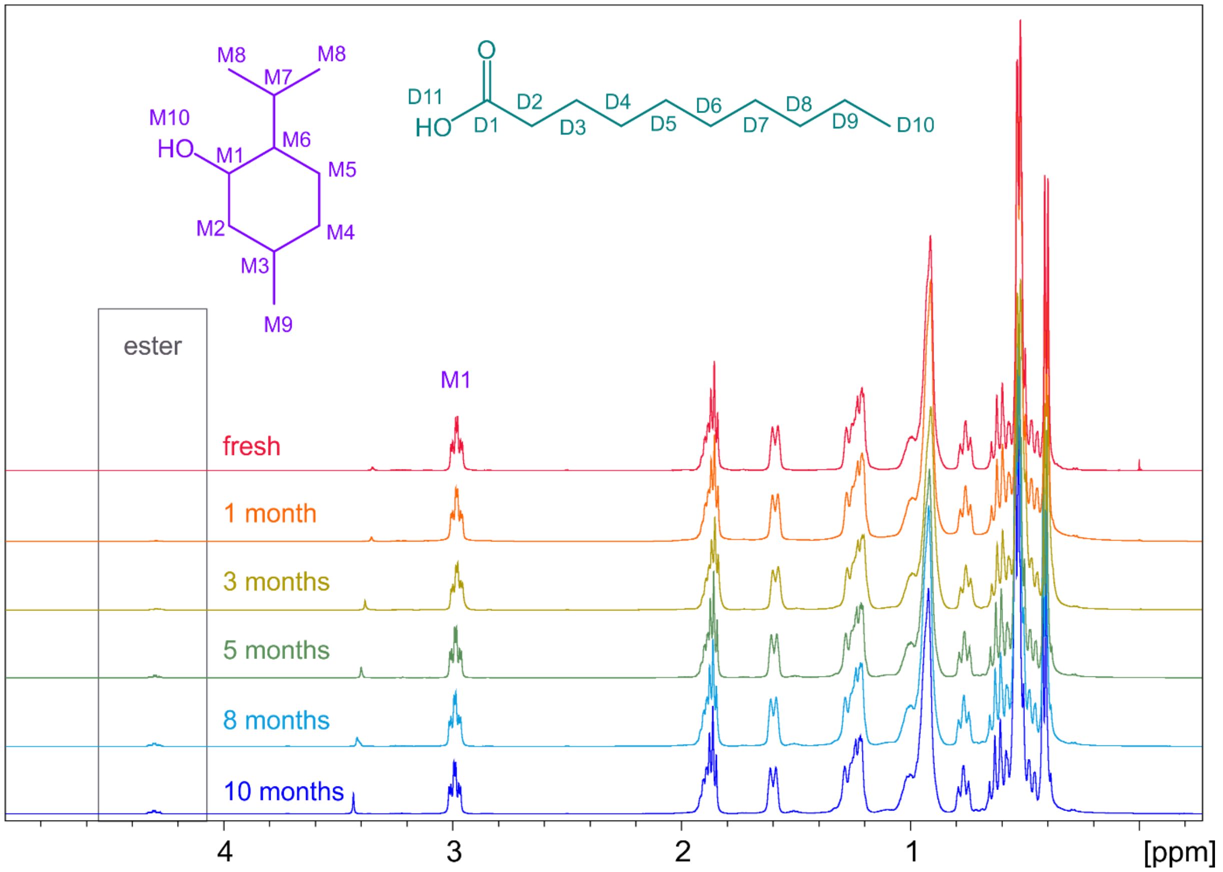

Figure S18. 1D ^{1}H NMR spectra at 25 °C of Men:DecA at different time after preparation.

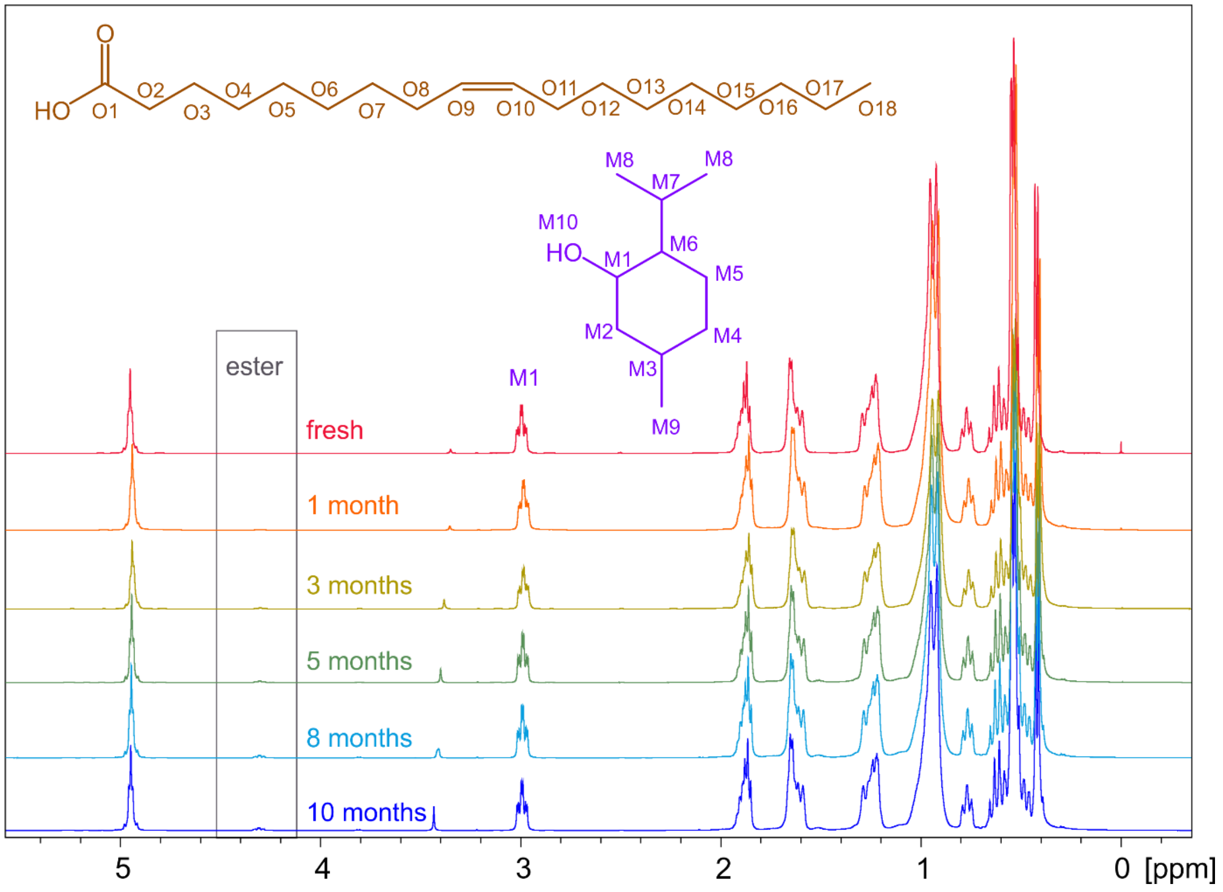

Figure S19. 1D ^{1}H NMR spectra at 25 °C of Men:OleA at different time after preparation.

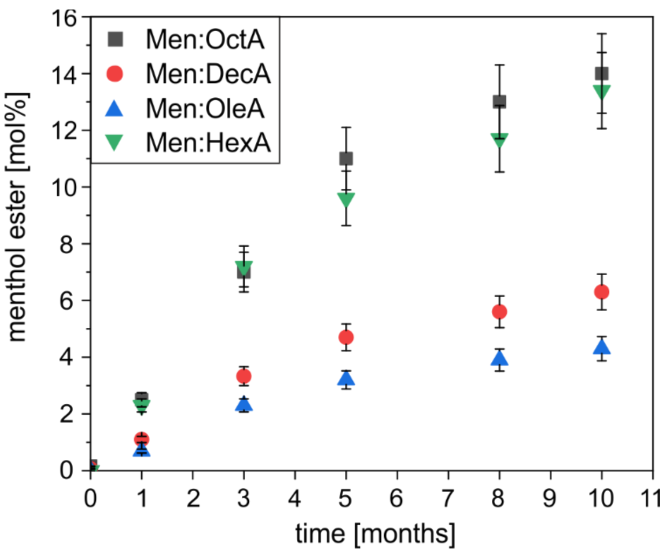

Figure S20. Relative concentration over time of menthol ester with respect to neat Men, as computed by integrating the signal at 4.3 ppm (assignable to M1 of the menthol ester) and the signal at 3.0 ppm (M1 of free Men) in the 1D ^{1}H NMR spectra at 25 °C of Men:HexA, Men:OctA, Men:DecA and Men:OleA with respect to time after preparation (in months).

Results of LLE experiments

Table S8. BPA extraction efficiency (EE), via NMR quantification, with relative standard deviation (rsd), and leaching of the Men component (Leach(Men)), using TOPO:Men in LLE under various experimental conditions.

Experiment	EE [%]	rsd	Leach(Men) [%]
1h - no stirring	59	15%	0.105±0.024
1h - stirring	>98.5	9%	0.100±0.021
10 min - stirring	>98.5	9%	0.075±0.012
10 min – stirring - no centrifugation	>98.5	9%	0.112±0.007

Table S9. Comparison of BPA concentration in the water and HES phase after LLE using TOPO:Men under various experimental conditions, as measured with UV-Vis and NMR spectroscopy.

Experiment	BPA concentration [ppm]		
	water phase (NMR)	water phase (UV-Vis)	HES phase (NMR)
1h - no stirring	46.5±4.9	36.9±2.1	59±9
1h - stirring	<1.5	21.0±8.6	>100
10 min - stirring	<1.5	7.8±5.7	>100
10 min – stirring - no centrifugation	<1.5	18.7±4.2	>100

Table S10. BPA extraction efficiency (EE), via NMR quantification, with relative standard deviation (rsd), and HES leaching (Leach(i), with i equal to Men in Men:OleA and TOPO:Men, and the average of the components for TOPO:DodA and OctA:DodA), using four different HES under the best extraction conditions (10min stirring and centrifugation).

Experiment	EE [%]	Rsd	Leach(i) [%]
TOPO:Men	>98.5	2.0%	0.075±0.012
TOPO:DodA	>98.5	2.0%	0.007±0.003
OctA:DodA	98	2.0%	0.113±0.007
Men:OleA	>98.5	2.0%	0.129±0.026

Table S11. Comparison of BPA concentration in the water and HES phase after LLE using four selected HES under the best extraction conditions (10min stirring and centrifugation), as measured with UV-Vis and NMR spectroscopy.

Experiment	BPA concentration [ppm]		
	water phase (NMR)	water phase (UV-Vis)	HES phase (NMR)
TOPO:Men	<1.5	7.8±5.7	>100
TOPO:DodA	<1.5	11.7±4.2	>100
OctA:DodA	1.8±1.8	15.1±2.2	>100
Men:OleA	<1.5	3.0±1.7	>100

Green metrics

Analytical EcoScale

Table S12. Penalty points (PP) introduced in the Analytical EcoScale [2] for the safety of the reagents.

	Number of pictograms	Symbol label	PPs
Men	1	warning	1
OctA	1	danger	2
DodA	1	danger	2
OleA	0	none	0
TOPO	1	danger	2

Table S13. Analytical EcoScale [2] evaluation in the scenario of optimized LLE protocol (10min stirring and centrifugation) using four selected HES, without HES recycling, with either UV-Vis or NMR quantification.

HES			TOPO:Men 1:2		TOPO:DodA 1:1		Men:OleA 2:1		OctA:DodA 3:1	
Conditions			UV-Vis	NMR	UV-Vis	NMR	UV-Vis	NMR	UV-Vis	NMR
**Reagents 1**	**Amount**	PPs								
	< 10 mL (g)	1	1	1	1	1	1	1	1	1
	10-100 mL (g)	2								
	> 100 mL	3								
**Reagent 2**	**Hazard (physical, environmental, health)**									
	None	0								
	less severe hazard	1	1	1			1	1		
	more severe hazard	2	2	2	2+2	2+2			2+2	2+2
**Reagent total PP**	**Amount PP x Hazard PP**		**3**	**3**	**4**	**4**	**1**	**1**	**4**	**4**
**Instrument 1**	**Energy**		**1**	**3**	**1**	**3**	**1**	**3**	**1**	**3**
	≤ 0.1 kWh per sample	0								
	≤ 1.5 kWh per sample	1								
	> 1.5 kWh per sample	2								

**Instrument 2**	**Occupational hazard**									
	Analytical process hermetization	0	**0**	**0**	**0**	**0**	**0**	**0**	**0**	**0**
	Emission of vapors and gases to the air	3								
**Instrument 3**	**Waste**									
	none	0								
	< 1 mL (g)	1								
	1-10 mL (g)	3	**3**	**3**	**3**	**3**	**3**	**3**	**3**	**3**
	> 10 mL (g)	5								
	recycling	0								
	degradation	1								
	passivation	2								
	no treatment	3	**3**	**3**	**3**	**3**	**3**	**3**	**3**	**3**
	Sum penalty points		10	12	11	13	8	10	11	13
	**Analytical EcoScale**		**90**	**88**	**89**	**87**	**92**	**90**	**89**	**87**

Table S14. Analytical EcoScale [2] evaluation in the scenario of optimized LLE protocol (10min stirring and centrifugation) using TOPO:Men, with HES recycling, with either UV-Vis or NMR quantification.

HES			TOPO:Men 1:2	
Conditions			UV-Vis	NMR
**Reagents 1**	**Amount**	PPs		
	< 10 mL (g)	1	1	1
	10-100 mL (g)	2		
	> 100 mL	3		
**Reagent 2**	**Hazard (physical, environmental, health)**			
	None	0		
	less severe hazard	1	1	1
	more severe hazard	2	2	2
**Reagent total PP**	**Amount PP x Hazard PP**		**3**	**3**
**Instrument 1**	**Energy**		**1**	**3**
	≤ 0.1 kWh per sample (UV-Vis, centrifuge)	0		
	≤ 1.5 kWh per sample (hot-plate)	1		
	> 1.5 kWh per sample (NMR)	2		
**Instrument 2**	**Occupational hazard**			
	Analytical process hermetization	0	**0**	**0**
	Emission of vapors and gases to the air	3		
**Instrument 3**	**Waste**			
	none	0		
	< 1 mL (g)	1	**1**	**1**
	1-10 mL (g)	3		
	> 10 mL (g)	5		
	recycling	0	**0**	**0**
	degradation	1		
	passivation	2		
	no treatment	3		
	Sum penalty points		5	7
	Analytical EcoScale		**95**	**93**

AGREEprep

The online emulator was accessed by following the link provided at the project's website (https://agreeprep.anvil.app/).

The pictogram consists of a coloured round pictogram with the final score in the centre, ranging from 0 (the least green achievable) to 1 (the greenest achievable). Ten trapezoid bars surround the central circle, representing the different criteria. Their length accounts for the weight of each step (default values are assigned, but it is possible to adjust them to the analytical goals, prior proper justification), while their colour and score account for the degree of sustainability (red and 0 for unsuitable conditions, to green and 1 for recommended conditions). The inputs used for the different criteria are justified as follows:

1) Favor in situ sample preparation: *ex situ sample preparation*. For real-world samples, collection and transport to the laboratory for sample preparation and analysis would be needed, which is the worst-case scenario and is assigned with the lowest score.
2) Use safer solvents and reagents: *0g for Men, DodA and OleA, the actual mass for OctA and TOPO*. According to their SDS, Men, DodA and OleA are considered safe, while OctA and TOPO are harmful to aquatic life and then ranked as problematic.
3) Target sustainable, reusable, and renewable materials:
    a. *> 75% of reagents and materials are sustainable or renewable*, for the scenario without recycling using HES composed of the terpene Men and the long-chain carboxylic acids OctA, DodA and OleA, which can be considered as sustainable as they "can be maintained for the foreseeable future without compromising future generations";
    b. *25-50% of reagents and materials are sustainable or renewable*, for the scenario without recycling using HES containing TOPO as precursor;
    c. *reagents and materials are not sustainable or renewable but can be used several times*, for the scenario with recycling using TOPO:Men.
4) Minimize waste:
    a. *9.1g-12.6g* in the scenario without HES recycling, with waste including: single-use glassware (8g-11.5g), reusable materials (0.1g), the HES (1g), regardless of its environmental, health, and safety issues;
    b. *8.2g* in the scenario with recycling of TOPO:Men, with waste including: single-use glassware (8g), reusable materials (0.2g).
5) Minimize sample, chemical and material amounts: *3g*. The actual sample size is considered.
6) Maximize sample throughput: *2 samples per hour*. No real evaluation has been done in terms of number of samples that can be prepared in one hour, and the estimate only considers the time needed for a triplicate during the experiments performed here.
7) Integrate steps and promote automation:
    a. *4 steps and manual system* in the scenario without HES recycling and UV-Vis quantification: HES preparation, extraction, centrifugation and separation were considered, all performed manually by an operator.
    b. *5 steps and manual system* in the scenario without HES recycling and NMR quantification: the NMR sample preparation is considered as an additional step.
    c. *≥ 6 steps and manual system* in the scenario with HES recycling: HES regeneration is considered additionally.
8) Minimize energy consumption: *< 10 Wh per sample*. Energy consumption was calculated considering the nominal power value of the electrical appliance used as listed in the manufacturer's technical

specifications sheet or on the back of the instrument's hardware (630W for hot-plate stirrer and 70W for centrifuge), properly scaled according to the specific time needed to complete the sample preparation step and then divided by the number of samples treated.

9) Choose the greenest possible post-sample preparation configuration for analysis: *0.75 for UV-Vis and 0 for NMR*. The two analytical techniques explored here contribute differently to the final score, with qNMR consistently causing a decrease by 0.5-0.6.

10) Ensure safe procedures for the operator: *1 hazards for OctA:DodA, Men:OleA and TOPO:DodA, and 2 hazards for TOPO:Men*. Men has a "health hazard pictogram" (GHS-07), while OctA, DodA and TOPO are corrosive (GHS-05), and OleA displays no pictogram. When the same hazard pictogram appears in the SDSs for more than one compound, it is recommended to count it only once. Therefore, all binary mixtures showed 1 hazard, except TOPO:Men (2 hazards).

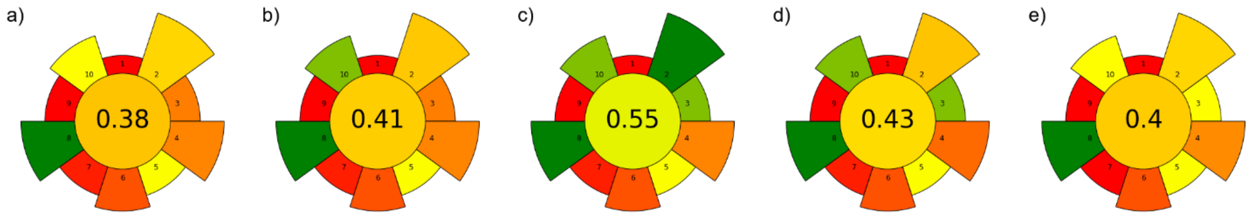

Figure S21. The result of AGREEprep [3] assessment of LLE procedure for BPA extraction using a) TOPO:Men 1:2, b) TOPO:DodA 1:1, c) Men:OleA 2:1, d) OctA:DodA 3:1, and e) TOPO:Men 1:2 with HES recycling, when NMR is used as analytical method.

References

[1] K. Van Aken, L. Strekowski, L. Patiny, EcoScale, a semi-quantitative tool to select an organic preparation based on economical and ecological parameters, Beilstein J. Org. Chem. 2 (2006) 1–7. https://doi.org/10.1186/1860-5397-2-3.

[2] A. Gałuszka, Z.M. Migaszewski, P. Konieczka, J. Namieśnik, Analytical Eco-Scale for assessing the greenness of analytical procedures, TrAC - Trends Anal. Chem. 37 (2012) 61–72. https://doi.org/10.1016/j.trac.2012.03.013.

[3] W. Wojnowski, M. Tobiszewski, F. Pena-Pereira, E. Psillakis, AGREEprep – Analytical greenness metric for sample preparation, TrAC - Trends Anal. Chem. 149 (2022) 116553. https://doi.org/10.1016/j.trac.2022.116553.
